# Optical control of NMDA receptors with a diffusible photoswitch

**DOI:** 10.1038/ncomms9076

**Published:** 2015-08-27

**Authors:** Laura Laprell, Emilienne Repak, Vilius Franckevicius, Felix Hartrampf, Jan Terhag, Michael Hollmann, Martin Sumser, Nelson Rebola, David A. DiGregorio, Dirk Trauner

**Affiliations:** 1 Department of Chemistry and Pharmacology, Ludwig-Maximilians-Universität, München, and Center for Integrated Protein Science, Munich 81377, Germany; 2 Institut Pasteur, Unit of Dynamic Neuronal Imaging, 25 rue du Dr Roux, Paris Cedex 15 75724, France; 3 CNRS UMR 3571, Genes, Synapses, and Cognition, Institut Pasteur, 25 rue du Dr Roux, Paris Cedex 15 75724, France; 4 Ruhr-Universität-Bochum, Department of Biochemistry, Bochum 44780, Germany

## Abstract

*N*-methyl-D-aspartate receptors (NMDARs) play a central role in synaptic plasticity, learning and memory, and are implicated in various neuronal disorders. We synthesized a diffusible photochromic glutamate analogue, azobenzene-triazole-glutamate (****ATG****), which is specific for NMDARs and functions as a photoswitchable agonist. **ATG** is inactive in its dark-adapted *trans*-isoform, but can be converted into its active *cis*-isoform using one-photon (near UV) or two-photon (740 nm) excitation. Irradiation with violet light photo-inactivates **ATG** within milliseconds, allowing agonist removal on the timescale of NMDAR deactivation. **ATG** is compatible with Ca^2+^ imaging and can be used to optically mimic synaptic coincidence detection protocols. Thus, **ATG** can be used like traditional caged glutamate compounds, but with the added advantages of NMDAR specificity, low antagonism of GABAR-mediated currents, and precise temporal control of agonist delivery.

## Abstract (web summary)

*N*-methyl-D-aspartate receptors (NMDARs) play a central role in synaptic plasticity, learning and memory. Here the authors describe azobenzene-triazole-glutamate (**ATG**), a new diffusible photoswitchable agonist that allows precise temporal control over NMDAR activity.

## Graphical abstract

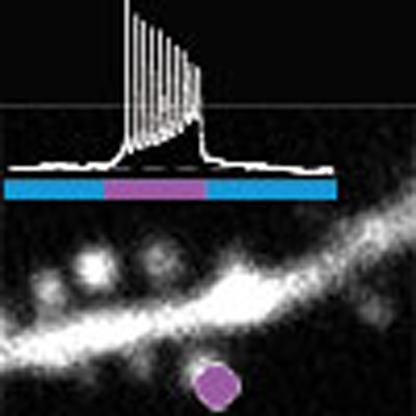

Ionotropic glutamate receptors mediate fast excitatory synaptic transmission and are ubiquitously expressed in the central nervous system. They can be separated into three major classes: α-amino-3-hydroxy-5-methyl-4-isoxazolepropionic acid receptors (AMPARs), kainate receptors and *N*-methyl-D-aspartate receptors (NMDARs). The latter are involved in the induction of synaptic plasticity, the cellular correlate of learning and memory^1^. NMDARs, of which a functional tetrameric receptor structure has been reported lately^2^,^3^, have also been implicated in a variety of neurological diseases and dysfunctions including ischaemia-related cell death, Alzheimer's, Huntington's and Parkinson's Diseases, as well as schizophrenia and autism spectrum disorders^1^. NMDARs are heterotetramers whose subunit composition varies based upon brain region, maturation and synaptic activity. Subunit differences result in variations in receptor kinetics, which confer different computational properties on the receptors^1^,^4^. Such variations in kinetic behaviour of NMDAR subunits have been examined on extrasynaptic and recombinant receptors using outside-out patch clamp recordings and fast application of agonist^5^,^6^.

The study of glutamate receptors in their native environment has been facilitated by the development of optical tools, namely light-sensitive caged agonists and antagonists, which take advantage of the temporal and spatial precision that light provides. Compounds including caged glutamate, caged NMDA and caged MK-801 have proven very useful for finely tuned, non-invasive studies of NMDARs^7^,^8^,^9^,^10^,^11^. For example, MNI-glutamate uncaging in a diffraction-limited laser illumination volume enables synaptic-like activation of both AMPARs and NMDARs^12^,^13^,^14^,^15^. Larger illumination volumes can be used to quantify activation and desensitization kinetics of synaptic receptors^15^. However, the study of deactivation of receptors must be done following rapid removal of agonist, a feat not possible with caged glutamate because of slow clearance by diffusion or uptake^15^. This is particularly challenging in functional networks within brain tissue.

Molecular photoswitches provide an interesting alternative to caged compounds since they can be rapidly and repeatedly switched on and off and do not generate photochemical byproducts. Over the past decade, several photoswitches have been developed including both photoswitchable tethered ligands and freely diffusible photochromic ligands (PCL) that allow for the optical control of transmembrane receptors and, by extension, neural systems^16^. **GluAzo** is a photochromic version of glutamate that functions as a PCL for the kainate receptors GluK1 and GluK2^17^, and **ATA**-3 is a PCL selective for AMPARs^18^ (Fig. 1a). Both **GluAzo** and **ATA**-3 are active in the thermally relaxed, dark-adapted state, thus their use with networks of neurons is challenging as the whole preparation must be illuminated to prevent tonic activity.

We now report a unique photochromic agonist that targets a different family of glutamate receptors. This compound, azobenzene-triazole-glutamate (**ATG**), complements **ATA**-3 and **GluAzo** since it selectively activates NMDARs (Fig. 1b). In addition, it possesses an important functional advantage that distinguishes it from our previously developed PCLs: it is inactive in the dark-adapted *trans*-isoform, but quickly converts into its active *cis*-isoform when irradiated with ultra-violet (UV) light (370 nm). As such, it is not excitotoxic when applied to neural networks in the absence of light, and it becomes an agonist of NMDARs with millisecond precision when irradiated. In addition, we show that **ATG** is the first diffusible PCL that can be precisely controlled with two-photon excitation (740 nm).

## Results

### Synthesis and Photophysical Characterization of **ATG**

**Figure 1:**
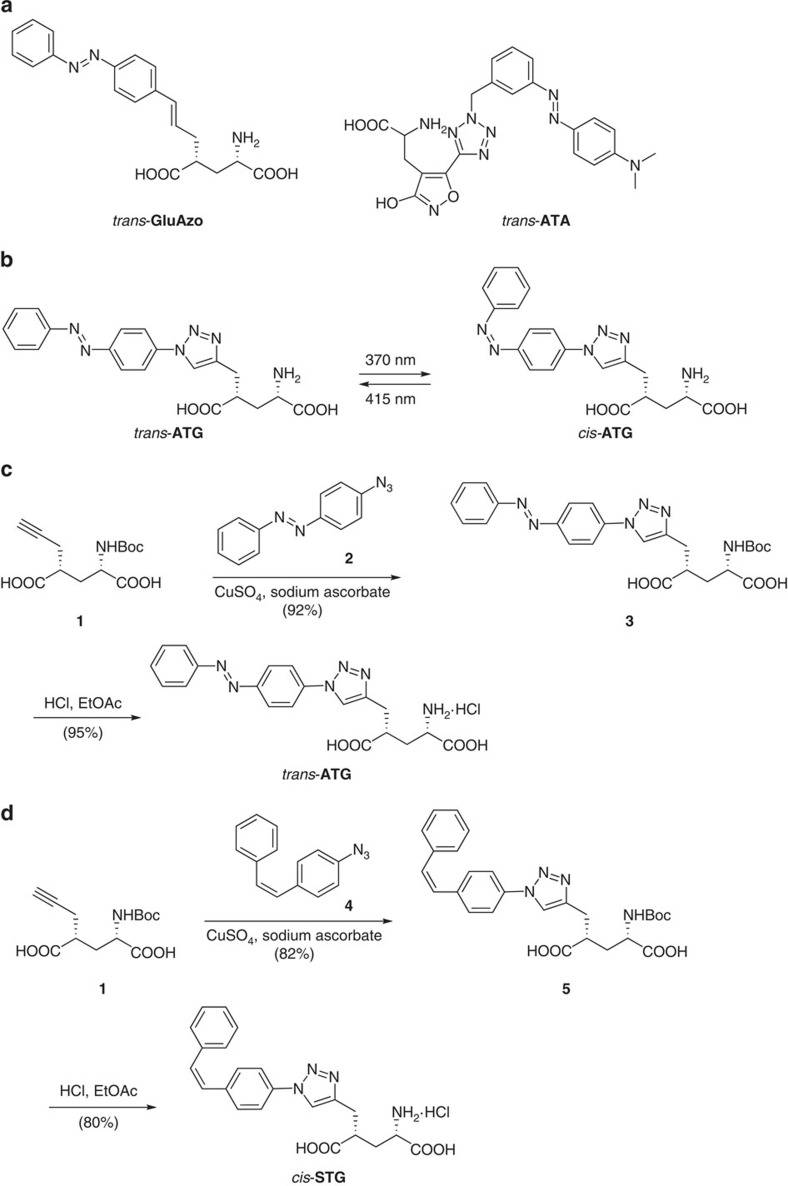
Design and synthesis of ATG. (**a**) Structures of **GluAzo**, a photochromic agonist of kainate receptors, and **ATA**, a photochromic agonist of AMPA receptors in their respective *trans* isoform. (**b**) Structure and photophysical properties of **ATG**. The molecule consists of a photoswitchable azobenzene, a triazole and a glutamate moiety. The *trans*- and *cis*-configuration of **ATG** are shown. (**c**) Synthesis of the azobenzene **ATG** using click chemistry. (**d**) Synthesis of the stilbene *cis*-**STG** using click chemistry.

**ATG** was synthesized in a few steps from the known glutamate derivative 4(*R*)-propargyl glutamate (**1**) using click chemistry (Fig. 1c). In brief, **1** was treated with azobenzene azide **2** in the presence of a copper catalyst to afford triazole **3**. Global deprotection then yielded **ATG**. A thermally stable stilbene analogue of *cis*-**ATG**, termed *cis*-**STG**, was synthesized in a similar fashion by treating **1** with **4** and deprotecting the resultant triazole **5** (Fig. 1d). Details of the synthesis and full characterization can be found in Supplementary Fig. 10, Supplementary Note 1 and in the Supplementary Methods.

**ATG** behaves as a regular azobenzene that can be converted to its *cis*-isoform with UV-A light. Conversion into the thermodynamically favourable *trans*-isoform requires irradiation with violet light (Fig. 1 and Supplementary Fig. 1a). Thermal relaxation into the *trans*-isoform is very slow in physiological buffer solution in accordance with the ‘regular azobenzene' nature of **ATG** (Supplementary Fig. 1b).

### Photopharmacology of **ATG**

**Figure 2:**
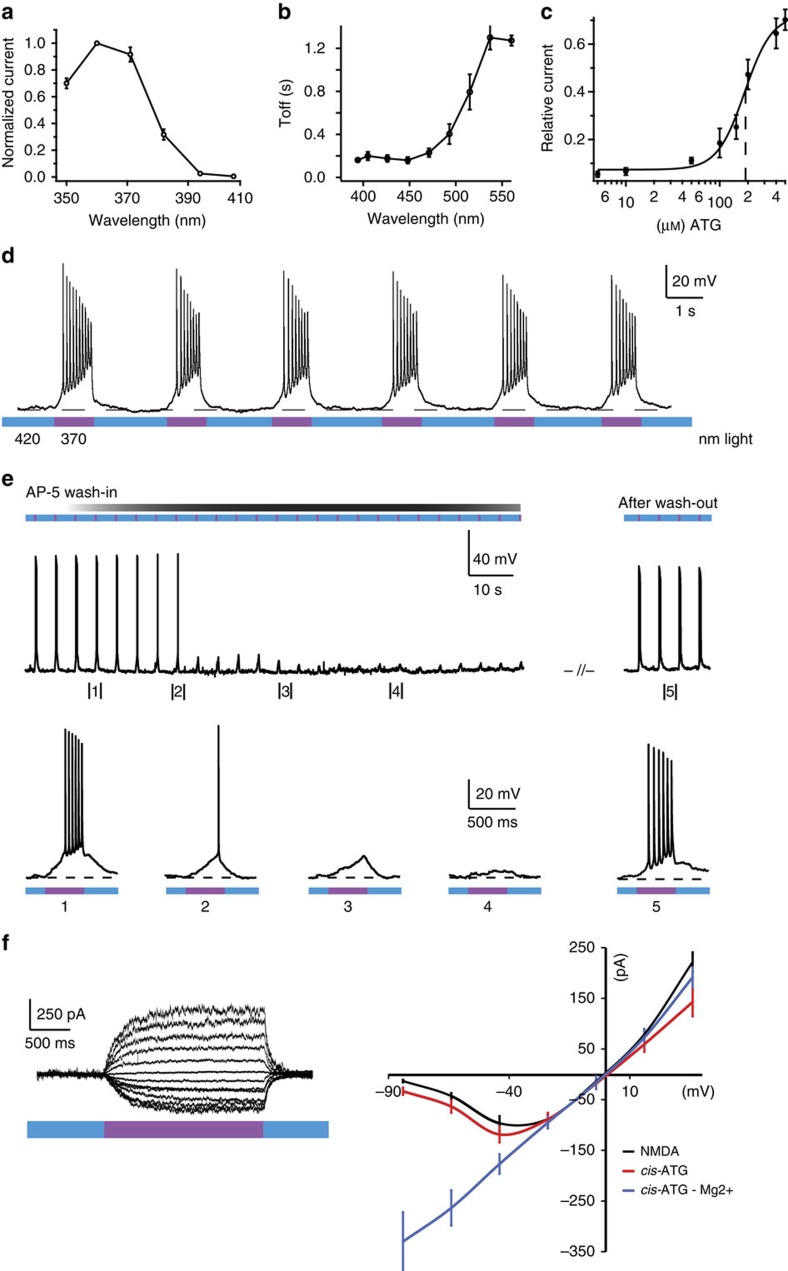
Photopharmacology of ATG. (**a**) Action spectrum of **ATG** recorded in layer 2/3 cortical neurons in an acute slice preparation in presence of 200 μM **ATG** in ACSF. Current amplitude was measured after 5 s light stimulation with the respective wavelength and normalized to the maximal current amplitude at 360 nm. (**b**) Wavelength screening for *τ*_off_ kinetics of **ATG**-mediated currents between 400 and 560 nm light. Best *τ*_off_ kinetics were achieved at 400–450 nm light. (**c**) Dose–response relationship of **ATG**-mediated currents in cortical slice preparations. Concentrations from 1 to 500 μM were tested. The EC_50_ is 185 μM (black dashed line) and was calculated using the Hill-equation. (**d**) Current-clamp recording of a layer 2/3 cortical neuron. Irradiation with 370 nm light (purple) induces robust action potential firing that is terminated by irradiation with 420 nm light (blue). (**e**) Washing in D-AP-5 (40 μM), an NMDA-specific antagonist, blocks the **ATG**-mediated light-dependent action potential firing. (**f**) Current–voltage relationships indicative of NMDARs as targets for **ATG**. Black; current–voltage relationship of puff-applied NMDA (200 μM) currents (*n*=12 cells). Red; current–voltage relationship of **ATG**-mediated currents under 370 nm light (*n*=10 cells). Blue; current–voltage relationship of **ATG**-mediated currents in the absence of Mg^2+^ ions (*n*=10 cells). Error bars indicate s.e.m.

To evaluate **ATG** as a photoswitchable agonist in neurons, we performed electrophysiological recordings in mouse layer 2/3 cortical neurons in acute coronal slices, while continuously perfusing 200 μM **ATG** in artificial cerebrospinal fluid (ACSF) (Fig. 2, Supplementary Fig. 2a,b). Using the whole-cell voltage-clamp configuration, we examined the spectral sensitivity of *cis*-**ATG**-evoked currents from 350 to 410 nm (Fig. 2a, Supplementary Fig. 2a). Maximal **ATG**-elicited currents were observed in response to 360 nm light, (3.75 mW mm^−2^; Supplementary Fig. 2c), whereas above 390 nm *cis*-**ATG**-mediated currents were negligible (<−10 pA). This action spectrum corresponds to the maximal conversion to *cis*-**ATG** after 365 nm irradiation, as determined by UV–VIS spectroscopy (Supplementary Fig. 1a). Switching back to *trans*-**ATG** was fastest using 425 nm light (*τ*_off_=0.17 s±0.03; *n*=16, Fig. 2b and Supplementary Fig. 2b). According to these data, **ATG** is *cis*-active, which is in sharp contrast to other photoswitchable glutamate receptor agonists previously developed^17^,^18^. Using our illumination system, the dose–response curve of **ATG** indicated an EC_50_ value of 185 μM under 370 nm light (Fig. 2c). In whole cell current-clamp recordings of mouse cortical layer 2/3 neurons, **ATG** triggered action potential (AP) firing under 370 nm light (Fig. 2d). Using 420 nm light, AP-firing could be quickly silenced. Thus, **ATG** photoswitching can be used to control neural activity with light.

We examined *cis*-**ATG**-mediated currents in the presence of various antagonists of ionotropic glutamate receptors (iGluRs) to identify the molecular targets of **ATG**. Application of NBQX (25 μM), an AMPAR-selective antagonist, had no effect on light-evoked currents (Supplementary Fig. 3). By contrast, D-AP5 (40 μM), a competitive NMDAR-selective antagonist, and MK-801 (50 μM), a use-dependent pore blocker that preferentially acts on NMDARs, completely abolished *cis*-**ATG**-mediated AP firing and currents, respectively (Fig. 2e and Supplementary Fig. 3)^19^.

Using heterologous expression strategies we next examined whether *cis*-**ATG** can activate NMDA receptors containing different isoforms. *Cis*-**ATG**-mediated currents were not detectable in HEK cells expressing different subunit combinations. In *Xenopus* oocytes, however, the high level of receptor expression facilitated the detection of *cis*-**ATG**-mediated currents (Supplementary Fig. 4). To normalize for different expression levels we compared the steady-state amplitude of *cis*-**ATG-**mediated currents in oocytes expressing recombinant diheteromeric NMDARs with those evoked by saturating NMDA concentrations (1 mM, Supplementary Fig. 4). We found that photoactivation of **ATG** is able to activate all subunit combinations between GluN1-1a and either GluN2A, B, C or D. The observation that *cis*-**ATG-**mediated currents were smaller than those elicited by superfusion of NMDA (<5%), may be because of increased light absorption resulting in incomplete photoconversion in the oocyte setup.

To further demonstrate the selectivity of **ATG** for NMDARs, we recorded current–voltage (I–V) relationships comparing NMDA (200 μM) puff application with **ATG** photoswitching (Fig. 2f). Because of their magnesium sensitivity, NMDARs are partially blocked at resting membrane potentials, imparting a J-shaped I–V relationship^20^ (Fig. 2f, right, black), which we observed for both NMDA application and *cis*-**ATG**-mediated currents (Fig. 2f). As expected for non-selective cation channels, the reversal potential was close to 0 mV. In the absence of external Mg^2+^ the I–V relationship was found to be linear, as expected for NMDARs (Fig. 2f, right, blue).

We further examined whether the thermally stable *cis*-**ATG** analogue (*cis*-**STG**, Fig. 1d), which does not photoswitch, has similar pharmacology and specificity for NMDARs. When puff applied, the stilbene *cis*-**STG** indeed elicited APs in mouse layer 2/3 cortical neurons (Supplementary Fig. 5a). The J-shaped I-V relationship (Supplementary Fig. 5b) indicates that *cis*-**STG** also targets NMDARs. As such, *cis*-**STG** represents a new structural class of agonist for these receptors.

One drawback of traditional caged glutamate compounds is that they are often antagonists of GABA_A_R-mediated synaptic currents^21^. We therefore tested the effect of *trans*-**ATG** on GABA_A_R-mediated inhibitory postsynaptic currents (IPSCs) in hippocampal CA1 pyramidal neurons. Using 400 μM bath application of **ATG**, we observed no detectable alteration of spontaneous IPSCs, but observed a 38±6% (*n*=10 cells) block of evoked IPSCs and an increase in coefficient of variation, consistent with a presynaptic target (Supplementary Fig. 6). Nevertheless, this is less than the 50% block by RuBi-glutamate (300 μM^21^), the 55% block by CDNI-glutamate (400 μM^22^) and the 83% block by the commonly used MNI-glutamate (300 μM^21^).

### ATG-mediated photoswitching of NMDAR gating

**Figure 3:**
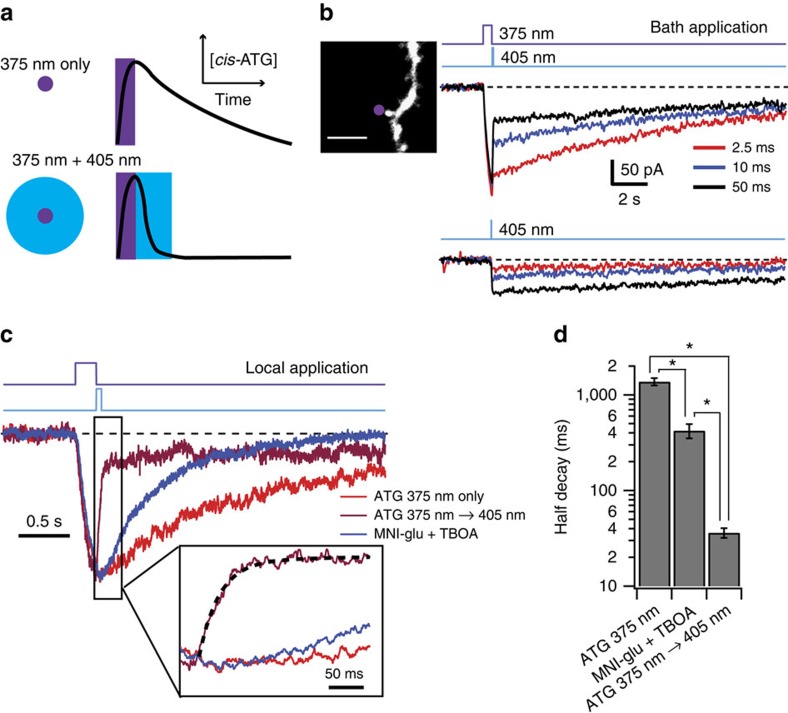
Dendritic NMDAR currents evoked by rapid laser-mediated photoswitching of ATG. (**a**) Schematic diagram showing putative sculpting of NMDAR gating by **ATG** photoswitching. A near-diffraction-limited spot of 375 nm light switches **ATG** to an active *cis*-conformation (top) that can activate the NMDAR transiently. When the 375 nm laser light is followed quickly by a brief 405 nm laser pulse focused over a larger volume, **ATG** is converted to the inactive *trans*-conformation (bottom), eliminating *cis*-**ATG**-mediated current more quickly than via diffusional clearance of *cis*-**ATG**. (**b**) Upper traces show light-evoked NMDAR currents recorded in CA1 pyramidal neurons while bath applying 200 μM **ATG**, in response to a 500 ms 375 nm laser pulse immediately followed by various durations of 405 nm laser pulses. Lower traces show smaller currents evoked by 405 nm pulses alone. Inset: confocal image of dendrite stimulated in these recordings. Purple dot indicates targeted point of **ATG** stimulation. Scale bar 3 μm (**c**) Normalized population averages of NMDAR currents evoked by 375 nm laser pulse (100 ms) alone (red; *n*=9 cells), or 375nm followed by 405 nm laser pulse (50 ms; magenta; *n*=9 cells) when locally applying **ATG** (100 μM) with a patch pipette. Blue trace represents uncaging-evoked NMDAR responses when locally applying MNI-glutamate (100 μM; *n*=5 cells). Dotted line on the magenta trace in the inset indicates the double exponential decay function. (**d**) Bar graph shows half-decay of NMDAR currents from cells in c. Error bars indicate s.e.m. *P<0.05 for all three comparisons (Steel Dwass all pairs nonparametric multiple comparison test).

We next considered the possibility that **ATG** photoswitching could be used to perform temporally precise agonism of NMDARs. We used fast digitally controlled diode lasers at 375 nm to switch to *cis*-**ATG** and 405 nm to preferentially switch to *trans*-**ATG** (Fig. 3a). The 375 nm laser light was focused to a near-diffraction-limited spot (full-width half-maximum (FWHM): 300 nm; Supplementary Fig. 7) and the 405 nm laser beam was adjusted to form a 4 μm spot (full-width half-maximum; Supplementary Fig. 7). NMDAR currents were recorded from CA1 pyramidal neuron dendrites within 100 μm of the soma at a holding potential of –30 mV. In the first set of experiments, 200 μM **ATG** was bath-applied (Fig. 3b). Laser illumination of 100 ms at 375 nm (∼150 μW) was required to see significant *cis*-**ATG**-mediated currents (average peak response: –87±12 pA; *n*=6 cells). Illumination for 5 s with 375 nm laser light resulted in large currents (–494±56 pA, *n*=18 cells) that rose over the light pulse duration (Supplementary Fig. 8a) and decayed over tens of seconds (Supplementary Fig. 8a,b). The reduction in *cis*-**ATG**-mediated current was maximal when using 50 ms illumination of 405 nm laser light (11 mW), but incomplete (56±3 % reduction, *n*=14 cells; Fig. 3b). The residual current was similar in amplitude to that when delivering a 50 ms 405 nm light pulse alone (*P*=0.35; Wilcoxon-matched pair signed rank test). The UV–VIS spectra indicate that irradiation with 405 nm light produces a small but measurable amount of *cis*-**ATG** (Supplementary Fig. 1a). This could explain why 405 nm illumination reduces current following 375 nm illumination, but evokes current when not preceded by 375 nm illumination (Fig. 3b).

When we examined the spatial dependence of *cis*-**ATG**-mediated currents, we saw little change in the peak amplitude even if the 375 nm laser was positioned up to 100 μm away from the dendrite. This finding, along with the slow rise and decay times, led us to the hypothesis that we had a very large effective photoactivation volume because of **ATG** photoswitching out-of-focus—an effect observed for bath application of caged compounds^23^. To further improve the kinetics of the NMDAR response, we tested local perfusion of **ATG** at 100 μM. Under these conditions, the decay of light-evoked *cis*-**ATG**-mediated currents was accelerated over sixfold, and we observed a halving of the peak current at only 5 μm away from the dendrite (Supplementary Fig. 8d). Local perfusion also permitted the use of higher concentrations of **ATG** to improve the activation time of NMDAR currents to <100 ms, similar to that of the widely used caged compound MNI-glutamate at similar concentrations (Supplementary Fig. 9a). Additionally, local application of **ATG** resulted in an enhanced fractional reduction of *cis*-**ATG**-mediated currents upon 405 nm light illumination (Fig. 3c and Supplementary Fig. 9b), up to 77±3% reduction, 100 μM (*n*=9 cells). Finally, and most importantly, local application of **ATG** resulted in faster decay of NMDAR currents following 405 nm illumination (**ATG**_off_): the half-decay of 33±4 ms (*n*=9 cells; Fig. 3d) was over 12 times faster than NMDAR current decays recorded in response to MNI-glutamate uncaging (422±72 ms, *n*=5 cells, *P*=0.01). Interestingly, the weighted decay time constant of **ATG**_off_ (*τ*_weighted_ of 102±42 ms; *n*= 9 cells; see example fit in Fig. 3c inset) was intermediate to the decay values for recombinant receptors containing either GluN1/GluN2A (*τ*_weighted_=29 ms) and GluN1/GluN2B (*τ*_weighted_=193 ms)^24^. We therefore considered the possibility that the rapid **ATG**_off_ decay could be used to estimate relative contributions of GluN2A and GluN2B to dendritic NMDAR activation.

**Figure 4:**
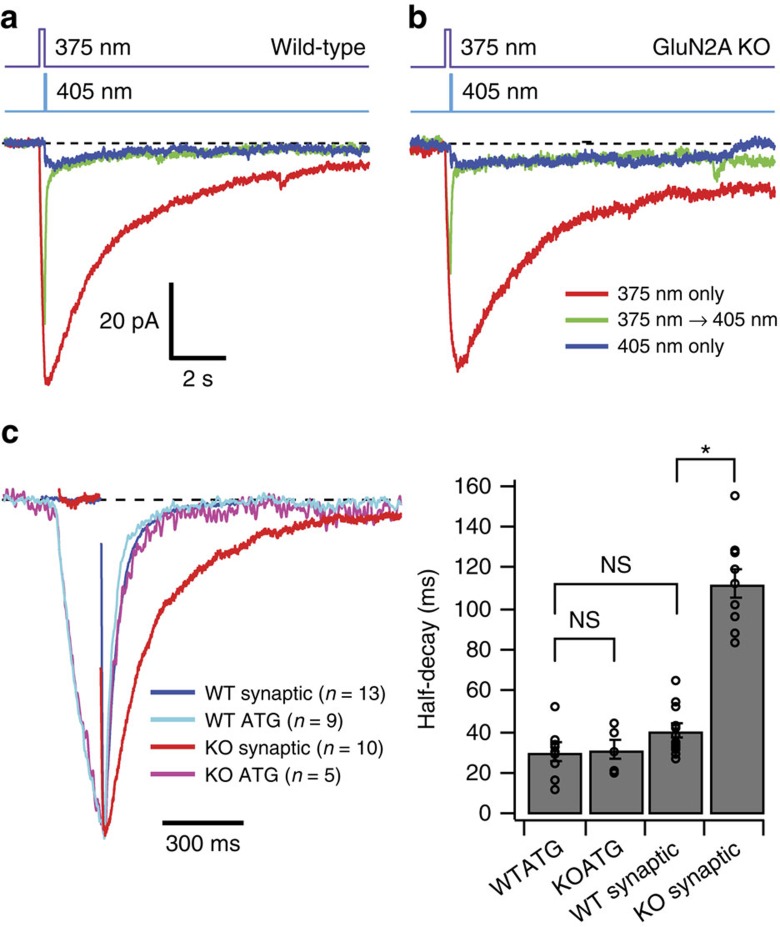
Comparison of ATG photoswitching responses between wild-type and GluN2A KO animals. (**a**) Population averages of light-evoked currents from WT CA1 pyramidal cells in response to 375 nm (100 ms) only, 375 nm followed by 405 nm (50 ms), and 405 nm only when locally applying **ATG** (100 μM) with a patch pipette (*n*=9 cells). (**b**) Population averages of photoswitching currents from GluN2A KO animals under same conditions as (**a**) (*n*=5 cells). **(c**) (left) Normalized currents in response to 375–405 nm photoswitching from (**a**) and (**b**) and population averages of NMDAR EPSCs in wild-type (*n*=13 cells) and KO animals (*n*=10 cells). Traces were aligned on their peaks and electrical artifacts from presynaptic stimulation have been blanked. Right: Bar graph of half-decays. Error bars indicate s.e.m. *P<0.05 and NS indicates comparisons that are not significantly different (Steel Dwass all pairs nonparametric multiple comparison test).

In mature CA1 pyramidal neurons (>P21), NMDARs are mostly comprised of tri-heteromers of GluN1, GluN2A and GluN2B^4^,^25^ whose kinetics are dominated by the rapid deactivation of GluN2A^26^,^27^. We therefore compared the decay of ****ATG****_off_ in WT and GluN2A KO mice. To get a better estimate of the **ATG**_off_ kinetics without contamination of 405 nm-induced *cis*-**ATG**-mediated currents, we subtracted a scaled curve fit to the 405 nm-induced current of each individual cell (Fig. 4a,b). The half-decay of subtracted **ATG**_off_ currents was 30±4 ms (*n*=9 cells) similar to the decay of NMDAR EPSCs recorded at the same age (40±3, *n*=13 cells; *P*=0.18; Steel Dwass all pairs nonparametric multiple comparison test; Fig. 4c). To determine whether the decay of **ATG**_off_ currents was influenced by GluN2A expression, we examined **ATG**_off_ decays in GluN2A KO mice (P29-P55). Surprisingly, the decay of **ATG**_off_ remained largely unaltered (KO half-decay: 31±5 ms, *n*=5, *P*=0.99 Steel Dwass all pairs nonparametric multiple comparison test), but the evoked synaptic responses were nearly three times slower (KO half-decay: 113±7 ms, *n*=10, *P*=0.0004, Steel Dwass all pairs nonparametric multiple comparison test; Fig. 4c), confirming the kinetic influence of GluN2A expression. Thus, the **ATG**_off_ decay is insensitive to subunit composition of NMDARs that are known to alter channel deactivation^28^.

### Two-photon activation of **ATG**

**Figure 5:**
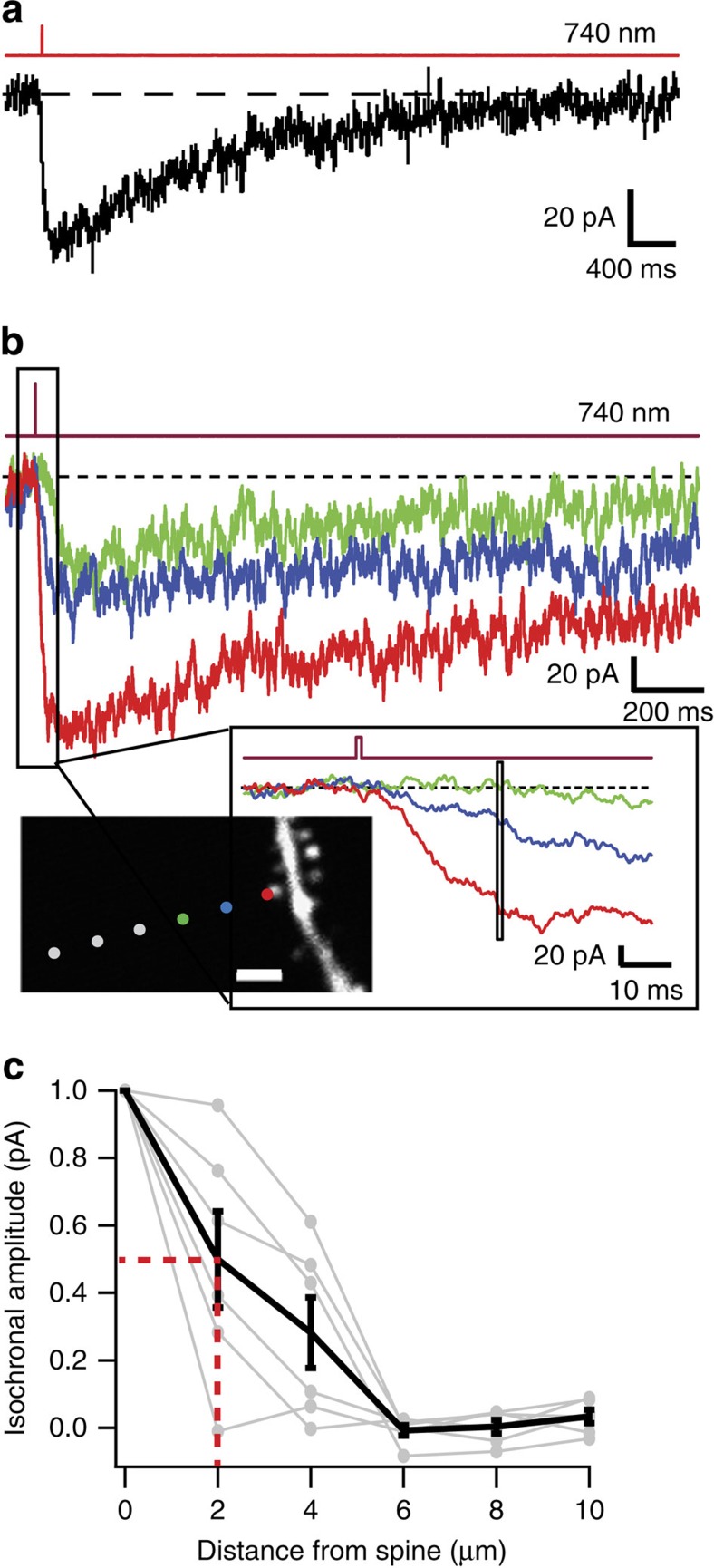
Localized two-photon activation of ATG. (**a**) *Cis*-**ATG**-mediated current evoked by two-photon illumination (1 ms, 740 nm) in a CA1 pyramidal cell while bath applying 400 μM **ATG**. (**b**) 2P-evoked *cis*-**ATG**-mediated currents with illumination spot parked at 0.5, 2 and 4 μm away from spine head. Illumination duration was 1 ms, and the wavelength set at 740 nm. (inset) Enlarged view of distance-dependent **ATG** evoked responses. Box over traces illustrates the time window over which spatial dependence was estimated for isochronal amplitude plots in (**c**) (Scale bar 2 μm). This was chosen to correspond to the time point at which the largest current reached 75% of its amplitude. (**c**) Normalized isochronal plots for six cells, with the average in black (half-width half-maximum=2.0 μm, red dotted lines). Error bars indicate s.e.m.

One strategy to achieve very localized photoactivation is to use two-photon (2P) excitation. We found that, during bath application of 400 μM **ATG**, femtosecond pulsed-laser illumination at 725–740 nm evoked detectable *cis*-**ATG**-mediated currents when using illumination durations as brief as 250 μs, which is much more efficient than one-photon activation of **ATG** (Fig. 5a), and similar to illumination durations required to evoke NMDAR currents using MNI-glutamate^29^,^30^,^31^,^32^. One millisecond duration pulses (740 nm) produced an average *cis*-**ATG**-mediated current of –48±7 pA (rise time (10-90%)=44±4 ms, *n*=16 spines), larger than the published values for single spine activation using caged-glutamate^29^,^30^,^31^. Nevertheless, spatial dependence of the amplitude of 2P-evoked *cis*-**ATG**-mediated currents indicated a local activation within 2 μm of the spine head (Fig. 5b,c).

### Combining **ATG** photoactivation with Ca^2+^ imaging

**Figure 6:**
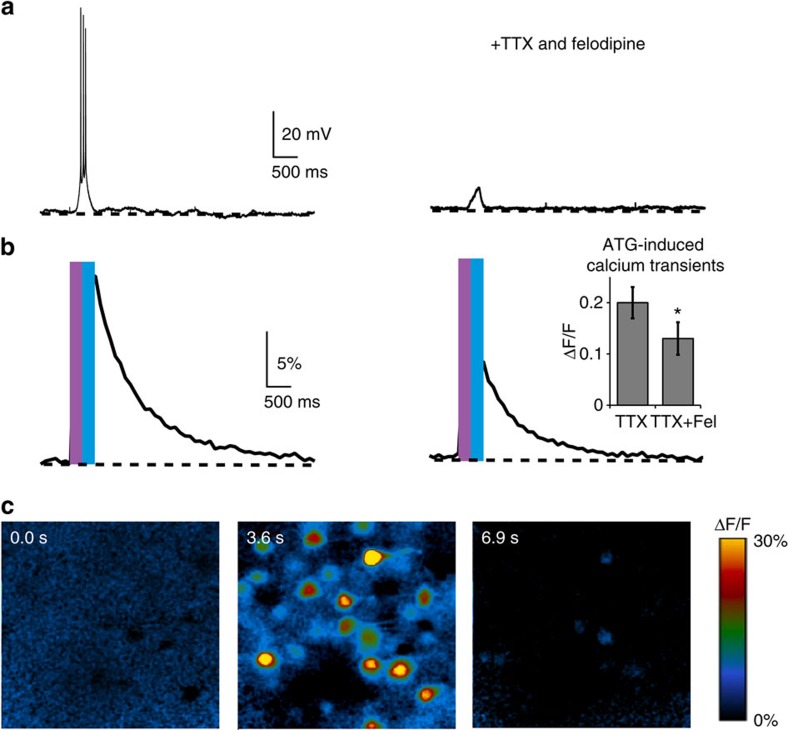
Calcium imaging using ATG in acute hippocampal slices. (**a**) *Cis*-**ATG**-mediated (200 μM) electrical signals in ACSF (left) and in the presence of 40 μM felodipine (Fel) and 1 μM TTX (right), elicited with 370 nm light and terminated with 420 nm (250 ms light pulse for each wavelength). (**b**) Calcium transients from responsive cells in the field of view corresponding to *cis*-**ATG**-mediated recording presented in (**a**). Bar graph: quantification of calcium transients (**ATG**+TTX: *n*=18 experiments and **ATG**+TTX+felodipine: *n*=10 experiments). *P<0.05, Wilcoxon rank-sum test. Error bars indicate s.e.m. (**c**) Changes in fluorescence (Δ*F/F*) at different time points of the calcium transient; prior to light stimulation, immediately after illumination and after returning to basal calcium levels.

The calcium permeability of NMDARs links their activity to postsynaptic biochemical alterations, such as spine morphology and glutamate receptor expression, which are associated with synaptic plasticity^20^. Since **ATG** specifically acts on NMDARs, we examined the possibility of imaging its effect with a Ca^2+^-sensitive fluorescent dye (Fig. 6). To this end, we incubated hippocampal slices with Quest Fluo-8-AM, a membrane-permeable Ca^2+^ indicator that is compatible with the activation wavelength of **ATG** because of its excitation wavelength of 490 nm (Fig. 6b). We applied tetrodotoxin citrate (TTX) (1 μM) and felodipine (40 μM), which prevents opening of voltage-gated Ca^2+^ channels to limit the response to NMDAR-mediated calcium entry (Fig. 6 right half). Indeed, upon irradiation with 370 nm in the presence of **ATG**, we observed light-evoked *cis*-**ATG**-mediated Ca^2+^ transients (Fig. 6b; Δ*F*/*F*=20±3%, *n*=18) which decreased in amplitude in the presence of blockers (Δ*F/F*=13±3%, *n*=10). We attributed the remaining Ca^2+^ transient to the influx through **ATG**-mediated NMDAR opening.

### Mimicking synaptic coincidence detection with ATG

**Figure 7:**
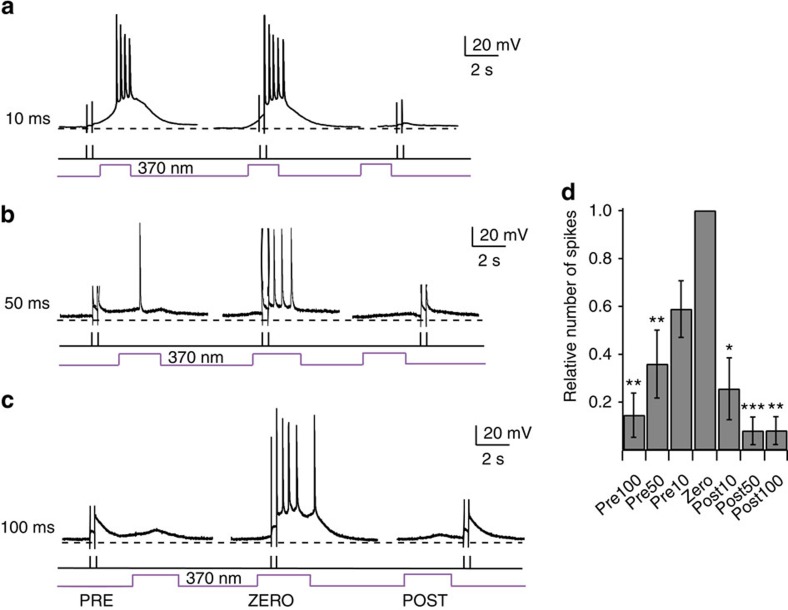
Coincidence detection using ATG in layer 2/3 cortical neurons. Coincidence detection of *cis*-**ATG** mediated current (200 μM) paired with antidromic stimulation. (**a**) Antidromic stimulation (black bars) of the postsynaptic cell 10 ms before, during and 10 ms after the light stimulation (purple trace). (**b**) As in (**a**), but with 50 ms intervals. (**c**) As in (**a**), but with 100 ms intervals. (**d**) Quantification of coincidence detection. Relative number of spikes compared with condition ZERO, when both stimuli were applied together (*n*=11 cells). Statistics were calculated using the Wilcoxon rank-sum test (*P<0.05,**P<0.01, ***P<0.001).

One of the most intriguing properties of NMDARs is their voltage-sensitive Mg^2+^ block at resting membrane potential. Accordingly, the presence of a neurotransmitter and concomitant postsynaptic depolarization are required to activate the ion channel, rendering NMDARs coincidence detectors of pre- and postsynaptic activity^33^. To test whether *cis*-**ATG**-mediated NMDAR activation can replace the synaptic stimulation necessary for coincidence detection, we designed a stimulus protocol, which couples antidromic stimulation (electrical stimulus of the axon hillock; Fig. 7, black line) of the postsynaptic cell with light activation (Fig. 7, purple line) of NMDARs at defined intervals. The electric stimuli were given at subthreshold intensities and did not induce AP firing by themselves. By varying the interval between the two stimuli, we showed that only coincident activation with light and current injection led to suprathreshold signals that generated APs. If the stimuli did not coincide or the delay between stimuli was too long, the number of spikes generated was significantly reduced (Fig. 7d).

## Discussion

To circumvent the limitations of traditional irreversible caged agonists, we synthesized a novel photo-reversible glutamate receptor agonist, **ATG**. This compound has functional features that make it unique and useful to neurobiological research: It is inactive in the dark, selectively targets NMDARs, can be quickly activated by irradiation with 360–375 nm light (or 725–740 nm light, 2P activation) and quickly deactivated with 405–460 nm light. Using rapid laser illumination, we demonstrated the unique photoswitching between a *cis*-active and *trans*-inactive compound, which enabled activation and deactivation of NMDARs on the timescale of their intrinsic gating properties. We also demonstrated that **ATG** is amenable to combination with other optical techniques, namely Ca^2+^ imaging. Finally, we showed that **ATG** could be used to mimic synaptic activation in coincidence detection protocols.

In the absence of detailed structural data and molecular dynamics calculations, it is difficult to explain why *cis*-**ATG** is the active form and why it is selective for NMDARs. Structure-activity relationship studies indicate that the pharmacological space available for **ATG** is relatively narrow, which makes it a challenge to develop red-shifted derivatives (D. Trauner, unpublished results). As the physiological activity of the stilbene analogue *cis*-**STG** indicates, the diazene unit (N=N bond) is not essential for ligand binding and is probably not engaged in hydrogen-bonding interactions to the ligand-binding domain. This is also apparent in a recent X-ray structure of GluAzo bound to the GluK2 ligand binding domain^34^. Attempts to red-shift the action spectrum of **ATG** by substituting it with strongly electron-donating substituents, such as a diethylamino group, in the 4′-position have so far yielded inactive compounds, indicating that substituents in this position clash with the ligand-binding domain in its closed state. Other ways to red-shift, for example, with heterocyclic azobenzenes, can be imagined and are under active investigation. Finally, *cis*-**ATG** and *cis*-**STG** represent a new class of agonists (and potentially antagonists) for ionotropic glutamate receptors that can be rapidly assembled using click-chemistry.

Herein, we show that **ATG** photoswitching and light-dependent NMDAR activation can be achieved with a monochromator, LEDs or laser light source. In principle, simple LEDs or standard light sources used in fluorescence imaging could also be used. It should be taken into account, however, that the kinetics of photoswitching are strongly dependent on the applied light intensities. **ATG** exhibits a high molar extinction coefficient (48,778 cm^–1^M^–1^ at 330 nm). Although such a feature enables efficient light absorption, it also reduces the light intensity at the focal point as a consequence of Lambert-Beer's law^23^. We circumvented this limitation by performing local perfusion of **ATG** to limit the optical path length in which photoswitching occurred. Two-photon excitation also provides a solution to the problem of out-of-focus light absorption while in parallel improving depth penetration in scattering tissue and providing intrinsic localization, properties well-suited for activation of single spines with a PCL. Deactivation at longer wavelengths can still be performed by large 1P illumination areas, which is advantageous for switching any molecules that have diffused out of the diffraction-limited illumination volume. Although the 2P cross-section has not been estimated, the ability of brief laser illumination durations (<1 ms) to evoke synaptic-like NMDAR current amplitudes could render **ATG** a useful complement to MNI-glutamate. Very recently, MAG_2p_ a derivative of glutamate that can be activated with 2P absorption, has been introduced^35^,^36^. This compound, however, is covalently attached to the receptor and not freely diffusible like **ATG**.

Because diffraction-limited illumination volumes are much larger than a single synaptic vesicle fusion event, the photolysis of caged neurotransmitter in diffraction-limited spots (by one- or two-photon excitation) is unlikely to mimic the rapid decay of neurotransmitter clearance^15^. PCL compounds, however, are ideally suited to overcome this diffusion-limited problem because photoisomerization of azobenzenes can occur on a picosecond timescale, sufficient for photo-inactivation of an agonist before it diffuses away. It remains to be determined if the switching speed is similar when *cis*-**ATG** is bound to the receptor. Nevertheless, we were able to demonstrate that **ATG** photoswitching can accelerate NMDAR current decays which, when using classical caged agonists, would have been limited by diffusional clearance. This requires that the inactive *trans-***ATG** have an affinity low enough to be effectively a non-agonist. Consistent with the relative low-affinity of *trans*-**ATG**, the **ATG**_off_ decays are nearly thirty times faster than the decay of *cis*-**ATG** currents. *Trans*-**ATG** also appears to unbind NMDARs more rapidly than glutamate, as the half-decay is over twelve times faster than that achieved by glutamate uncaging (Fig. 3). We demonstrate, for the first time, the fundamental ability to switch ‘off' channel agonism, thereby speeding the decay of NMDAR currents.

We were surprised that the **ATG**_off_ decay was not altered in GluN2A KO mice, especially since faster unbinding rates of glutamate are thought to underlie the faster deactivation rates of GluN2A-containing channels^34^. One possible interpretation of these results is that the affinity of *trans*-**ATG** is so low that photoconversion reveals a ligand-independent closing transition. To confirm this, future experiments would be required, especially to confirm rapid switching of *cis*-**ATG** when bound to the NMDAR.

Our results show that **ATG** is a powerful tool that permits precise temporal control of NMDAR gating not otherwise achieved with state-of-the-art caged compounds. They extend the reach of photopharmacology to an important subtype of glutamate receptors and demonstrate that photoswitchable neurotransmitters that are inactive in the dark can be synthesized and used to precisely control receptor activation in their native environment.

## Methods

### UV–Vis spectra

**ATG** was dissolved to a concentration of 50 μM in buffer containing (in mM) 138 NaCl, 1.5 KCl, 2.5 CaCl_2_, 1.2 MgCl_2_, 10 Glucose and 5 HEPES, adjusted to pH 7.4. UV–VIS spectra were taken in a 100 μl cuvette with the switching light (monochromator) introduced through a glass fibre from the top of the cuvette, perpendicular to the light path of the spectrometer (Varian, Cary 50). The kinetics of the *trans-* to *cis-*conversion were recorded at the maximal absorption wavelength of *trans*-**ATG** (330 nm). To achieve fast switching rates, we used high power LEDs at 365 and 460 nm (Prizmatix) for *trans-cis* and *cis-trans* isomerization, respectively.

### Cortical slice preparation and external solutions

Cortical coronal slices were prepared from C57Bl6JRj mice (postnatal day 10–15, both male and female animals were used without known experimenter bias). Following decapitation, the brain was rapidly removed and transferred to an ice-cold saline solution composed of (in mM) 2.5 KCl, 1.25 NaH_2_PO_4_, 25 NaHCO_3_, 0.5 CaCl_2_, 7 MgCl_2_, 25 glucose, 75 sucrose saturated with carbogen (95% O_2_/5% CO_2_). Slices (300 μm thick) were made using a Campden vibratome 7,000 smz-2 (NPI Electronic). Slices were incubated at 34 °C for 30 min in ACSF composed of (in mM) 125 NaCl, 2.5 KCl, 1.25 NaH_2_PO_4_, 26 NaHCO_3_, 2 CaCl_2_, 1 MgCl_2_, 20 glucose saturated with carbogen (95% O_2_ and 5% CO_2_). After incubation, slices were stored at room temperature from 30 min to five hours before being recorded. Experiments were carried out at room temperature. Unless stated otherwise, **ATG** was added from a 200 mM dimethyl sulfoxide stock to the ACSF to yield a final concentration of 200 μM. The ACSF was heated to 40 °C to improve the solubility of the **ATG** stock. The solution was not filtered because **ATG** adheres to filter materials.

For the identification of target receptors and calcium imaging experiments, the iGluR antagonists NBQX (25 μM) and D-AP-5 (40 μM), and the channel blockers TTX (1 μM) and felodipine (40 μM) were bath-applied, whereas MK-801 (50 μM) (all from Abcam) was loaded into the patch pipette. NMDA (1 mM, Sigma-Aldrich) and *cis*-**STG** (200 μM) were puff-applied through a glass pipette using a pressure ejection system (PDES, NPI Electronic). For voltage-clamp recordings, TTX was added to the ACSF.

### Patch clamp recordings of cortical layer 2/3 neurons

Pyramidal neurons were patched using fire-polished glass electrodes with a resistance of 6–9 MΩ. Current-clamp recordings were carried out using the following intracellular solution (in mM): 140 K-gluconate, 10 HEPES, 12 KCl, 4 NaCl, 4 Mg-ATP, 0.4 Na_2_-GTP. For whole-cell voltage-clamp recordings, we used (in mM) 110 Cs-gluconate, 15 NaCl, 10 HEPES, 5 TEA, 0.16 EGTA, 4 Mg-ATP, 0.4 Na_2_-GTP. Recordings were made with an EPC 10 USB amplifier, controlled by the Patchmaster software (HEKA). Data was filtered at 2.9–10 kHz and digitized at 50 kHz. Holding potential was corrected for a 14 mV liquid junction potential. Cells were rejected if leak currents were >200 pA or series resistance >25 MΩ. Data was analysed using the Patcher's Power Tools (MPI Göttingen) and routines written in IgorPro (Wavemetrics).

For antidromic stimulation, glass electrodes (5 MΩ) filled with ACSF were placed within 20 μm of the axon hillock and the stimulus pulse was applied through an isolated stimulation unit (A-M Systems). The stimulation intensity was set to be subthreshold. The temporal pattern of the antidromic and **ATG** light stimuli were controlled through the Patchmaster software (HEKA).

### Hippocampal slice preparation and external solutions

Hippocampal coronal slices were prepared from C57Bl6JRj mice (Janvier Labs) (postnatal day 15-55, both male and female animals were used without known experimenter bias) following decapitation and rapid removal of the brain^37^. A Leica VT1200S vibratome was used to make 250 μm thick slices while the brain was immersed in an ice-cold saline solution composed of (in mM) 2.5 KCl, 1.25 NaH_2_PO_4_, 25 NaHCO_3_, 0.5 CaCl_2_, 8 MgCl_2_, 25 glucose, 230 sucrose and 0.5 ascorbic acid saturated with 95% O_2_/5% CO_2_. After 30 min incubation at 33 °C in solution composed of (in mM) 125 NaCl, 2.5 KCl, 1.25 NaH_2_PO_4_, 25 NaHCO_3_, 2 CaCl_2_, 1 MgCl_2_, 25 glucose and 0.5 ascorbic acid saturated with 95% O_2_/5% CO_2_, slices were stored at room temperature from 30 min to 5 h in the same solution before being recorded. NMDAR currents were recorded in the presence of (in μM) 10 SR95531 (Abcam Biochemicals) to block GABARs, 0.3 strychnine (Sigma-Aldrich) to block glycine receptors, 5 2,3-Dioxo-6-nitro-1,2,3,4-tetrahydrobenzo[*f*]quinoxaline-7-sulfonamide (NBQX) (Abcam Biochemicals) to block AMPARs, and 1 TTX (Abcam Biochemicals) to minimize spontaneous activity. 50 μM D-serine (Sigma-Aldrich) was also included in the bath solution to saturate the co-agonist binding site of NMDAR. GABAR currents were isolated through the addition of (in μM) 0.3 strychnine, 5 NBQX, 10 D-(-)-2-Amino-5-phosphonopentanoic acid (Abcam Biochemicals) and 20 7-chlorokynurenic acid (Abcam Biochemicals). **ATG** or 4-methoxy-7-nitroindolinyl-caged L-glutamate (MNI-glutamate; Tocris Bioscience) was perfused locally using a 3–6 μm tip-diameter patch pipette. The **ATG** and MNI-glutamate perfusion solutions contained (in mM) 110 NaCl, 2.5 KCl, 2 NaHCO_3_, 1.25 NaH_2_PO_4_, 30 HEPES, 10 Glucose, 2 CaCl_2_, 1 MgCl_2_, 0.05 Alexa Fluor 488 (Life Technologies), 0.01 SR95531, 0.0003 strychnine, 0.005 NBQX, 0.001 TTX, 0.05 D-serine, and where noted, 0.05–0.1 D, L-*threo*-β-Benzyloxyaspartic acid (Tocris Bioscience, Bristol, UK). Alexa Fluor 488 was used to visualize the perfusion and ensure its regularity over the course of the experiment. The pH of the final perfusion solution was adjusted to 7.3 after dilution. As **ATG** is not readily soluble in water, a stock solution (10 mM) was prepared in 0.1N NaOH.

### Patch clamp recordings in hippocampal CA1 pyramidal neurons

Whole-cell voltage-clamp was performed from visually identified hippocampal CA1 pyramidal cells at 32 °C using an Axopatch 700B, Molecular Devices. Fire-polished patch electrodes had a tip resistance of 4-6 MΩ, and contained a Cs-methanesulfonate-based internal solution composed of (in mM) 105 CsCH_3_O_3_S, 10 EGTA, 3 CaCl_2_, 4 MgCl_2_, 10 Hepes, 4 NaCl, 4 NaATP, 0.4 NaGTP and 5 phosphocreatine for recording NMDAR currents. To record GABA_A_R currents, we used a similar solution except that we did not use NaCl and we added (in mM) 70 CsCl and 35 CsCH_3_O_3_S. 40-100 μM Alexa Fluor 594 was also added to the solution to visualize dendritic morphology during whole-cell experiments for all experiments except GABA_A_R recordings. Extracellular stimulation of EPSCs and IPSCs was performed at 0.1 Hz using a constant voltage stimulator (20–60 V, 50 μs, Digitimer) and a 4 MΩ resistance pipette. We recorded IPSCs for 10 min in ACSF, then 20 min in ACSF plus 400 μM **ATG**. Currents were filtered at 4–10 kHz then digitized at 100 kHz (NI PCI-6052E, National Instruments) using the software Neuromatic (www.neuromatic.thinkrandom.com/). Offline, traces were filtered between 4–10 kHz. Holding potentials were corrected for a −7 mV liquid junction potential (JPCalcW), and then set at −30 mV, except for GABA_A_R currents which were recorded at −70 mV.

### Photoswitching of **ATG** and calcium imaging

A Poly-V monochromator (FEI Systems) controlled through the Patchmaster software was used to toggle between *trans*- and *cis*-**ATG** at varying wavelengths (370 and 420 nm unless otherwise indicated). During calcium imaging experiments, the monochromator was controlled by the Live Analysis software (FEI). The calcium indicator Quest-Fluo-8-AM (50 μM MoBiTec) was added to the ACSF, and acute hippocampal slices were incubated for at least 20 min at 37 °C to facilitate uptake of the indicator. Calcium changes were recorded at 480 nm, digitized at 10 Hz, background corrected and the Δ*F/F*_0_ ratio was calculated using IgorPro routines and ImageJ (NIH).

### Laser-mediated photoswitching and uncaging

Visually guided patch experiments were performed using differential interference contrast or Dodt contrast. Dendrites were visualized using either a confocal (592 nm excitation of Alexa Fluor 594) or a 2P imaging system (810 nm, Ultima, Prairie Technologies). We coupled a 405 nm diode laser (Model PhoxX 405-120, Omicron Laserage) and a 375 nm diode laser (Model PhoxX 375, Omicron Laserage) into the photoactivation galvanometers of the scanhead (for rapid spot positioning) using single-mode optical fibres (Part QPMJ-A3S,A3S-400-3/125-3-2-1, Oz Optics). For one-photon photoswitching, we used 150 μW (bath application) or 375 μW (local application) of 375 nm and 10.5 mW of 405nm, measured after the objective. For two-photon photoactivation, a pulsed Ti:Sapphire laser (Chameleon Ultra II, Coherent) tuned to either 725nm or 740 nm was directed into a photoactivation path. Photoactivation was performed on proximal dendrites within 100 μm of the soma to minimize possible effects of dendritic filtering. Illumination spots were typically placed <0.5 μm from the tip of the spine head, except when longer duration pulses were used (>500 ms), in which case the spot was placed 1 μm away.

### *In vitro* transcription and preparation of cRNA

For expression in *Xenopus laevis* oocytes, clones (derived from *Rattus norvegicus*) of GluN1-1a (genebank accession number: U08261), GluN2A (AF001423), GluN2B (U11419.1), GluN2C (U08259.1) and GluN2D (U08260.1), each in the *X. laevis* oocyte expression vector pSGEM, were transcribed to cRNA *in vitro* using the T7 mMESSAGE mMACHINE Kit (Ambion) according to the protocol provided. Transcribed cRNA was isolated with a spin-column kit (Clean & Concentrator 25, Zymo) and cRNA integrity was checked via denaturing agarose gel electrophoresis. RNA concentration was determined photometrically with a NanoPhotometer (Implen) and the concentrations of all samples were adjusted to 200 ng μl^−1^ with nuclease-free water.

### Expression and two-electrode recordings in *Xenopus* oocytes

Frog oocytes were surgically removed from the ovaries of *X. laevis* (Nasco) anesthetized with ethyl 3-aminobenzoate methanesulfonate (2.3 g l^−1^; Sigma). The lumps of oocytes were incubated with 300 U ml^−1^ (10 mg ml^−1^) collagenase type I (Worthington Biochemicals) for 3 h at 21 °C in Ca^2+^-free Barth's solution (in mM) 88 NaCl, 1.1 KCl, 2.4 NaHCO_3_, 0.8 MgSO_4_, 15 HEPES, pH adjusted to 7.6 with NaOH) with slow agitation to remove the follicular cell layer, and then washed extensively with Barth's solution (in mM) 88 NaCl, 1.1 KCl, 2.4 NaHCO_3_, 0.8 MgSO_4_, 0.4 CaCl_2_, 0.3 Ca(NO)_3_ 15 HEPES, pH adjusted to 7.6 with NaOH). Oocytes were maintained in Barth's solution supplemented with 100 μg ml^−1^ gentamycin, 40 μg ml^−1^ streptomycin and 63 μg ml^−1^ penicillin. Intact oocytes of stages V or VI were selected and cRNA was injected with a Nanoliter 2010 injector (WPI) within 8 h after surgery. For expression of GluN1/GluN2 heteromers, 20 nl (4 ng) of cRNA for each subunit were injected. Electrophysiological recordings were carried out 5 days after injection. Two-electrode voltage clamping was performed using a TurboTec-10CX amplifier (npi electronic) controlled by Pulse software (HEKA). For photoswitching experiments, the recording chamber was illuminated using LEDs (365 and 460 nm, Prizmatix) coupled to a light guide, which was placed directly above the oocyte. LEDs were controlled via the TTL outputs of the ADC/DAC (ITC-16, Instrutech). Borosilicate glass capillaries (Harvard Instruments) were pulled to resistances of 0.1–1 MΩ with a vertical puller (PIP5, HEKA) and filled with 3 M KCl.

Oocytes were clamped at −70 mV. All recordings were performed in continuously superfused with Barium Ringer (BaR, in mM) 115 NaCl, 2.5 KCl, 1.8 BaCl_2_, 10 HEPES-NaOH, pH 7.2). When a stable holding current was attained, the recording protocol was started. To further prevent opening of calcium-induced chloride channels, niflumic acid (NFA, 250 μM) was added to the BaR. All agonist solutions contained 10 μM glycine. In addition, the BaR used for recording photo-currents was supplemented with **ATG** (200 μM). For recording of *cis*-**ATG** mediated currents, the microscope light was switched-off and the photoswitching recording sequence was started. A 5 s pulse of blue light (460 nm) was followed by 5 s of UV light (365 nm) and, again, 5 s of blue light. These protocols were carried out first in the absence of **ATG** to control for possible artifacts, and then repeated in the presence of 200 μM **ATG**. For the recording of NMDA-induced currents, 1 mM NMDA in BaR supplemented with 10 μM glycine was perfused. After steady-state currents were achieved, the NMDA was washed out until a stable baseline was reached. The application of 1 mM NMDA was repeated until two consecutive applications resulted in similar steady-state amplitudes. For analysis, light-induced **ATG**-independent currents were subtracted from *cis*-**ATG**-mediated currents. The steady-state amplitude of the corrected *cis*-**ATG**-mediated currents were then normalized to steady-state amplitude of NMDA-induced currents.

### Data analysis

Data analysis was performed using the Neuromatic analysis package and custom routines within the IgorPro environment (Wavemetrics). Cells were rejected from analysis if the leak current was >−100 pA at −30 mV holding potential or the series resistance was over 15 MΩ, except for the GluN2A KO animals in which larger leak currents were accepted. *Cis*-**ATG**-mediated current peak amplitudes from one-photon photoswitching were measured over a 1 ms window around the peak. Error bars are presented as the mean±s.e.m. unless otherwise indicated. Current decays were estimated from a fit with a double exponential equation *y*=*A*_1_exp{−(*x*−*x*_0_)}/*τ*_1_}+*A*_2_exp{−(*x*−*x*_0_}/*τ*_2_}. The weighted decay time constant (*τ*_weighted_) was calculated as 
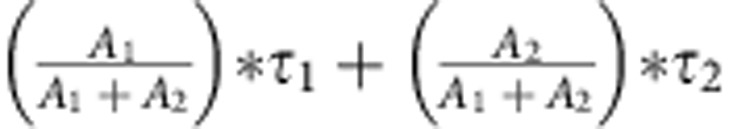
. Isochronal amplitudes represent averages of the currents in a 200 μs window centred at the time point at which the on-spine response had reached 75% of its peak value.

To better estimate the NMDAR current decay following 405 nm illumination without contamination from partial *cis*-activation of **ATG**, we performed a subtraction protocol. 405 nm-induced currents were fit to an empirical function that describes the rising phase and dual exponential decay^38^:


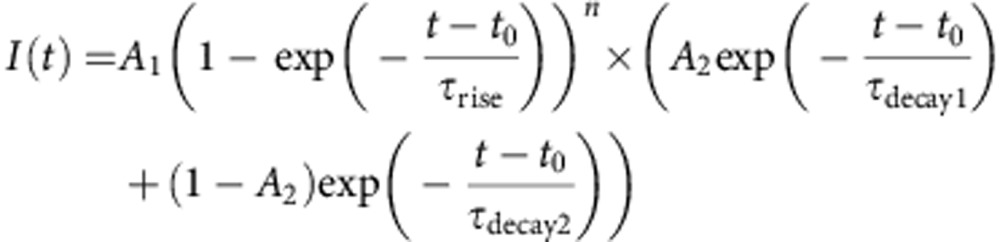


The fits of each individual cell were scaled by eye to maximally overlay the slow decay component of 405 nm only and 375/405 nm-induced currents. We justified such a scaling because the population traces overlapped (Fig. 4a,b). The scaled fits for each cell were then subtracted from the associated average current trace induced by 375 nm illumination followed by 405 nm illumination. Cells were only analysed if three or more recordings were performed under each illumination condition (375 nm only, 375 nm followed by 405 nm, 405 nm only).

GABAR spontaneous events (Supplementary Fig. 6) were detected using an event detection algorithm within the Neuromatic analysis software based on initial 10 pA threshold detection. Events were refined using two additional search features: (1) event onset detection using a 1.5 ms backward sliding window to ensure the point of threshold detection was 7 × s.d. of background noise; and (2) event peak detection using a 0.2 ms forward sliding window to ensure the event peaked within 2 ms of the time point of event threshold detection with a current greater than that of the point of event threshold by at least 2 × s.d. of background noise. Parameters were determined empirically, but were held constant for all analysis.

Peak amplitudes of IPSCs (Supplementary Fig. 6) were estimated from a 200 μs window centred at the time point of the peak value of the average of all events for a particular condition (number of events were typically between 50 and 300 per cell). Each synaptic current peak amplitude was corrected for series resistance error, as well as for a slight (∼5%) decay in peak amplitude of IPSCs over the duration of the recording, which was not due to **ATG** activity (estimated from sham experiments, *n*=5 cells). The coefficient of variation of the GABAR IPSCs was calculated as 
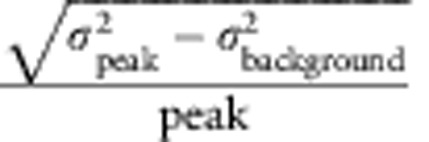
 where *σ* represents the s.d. over a 200 μs window. Background variance was measured as the standard deviation over a 200 μs window immediately before the stimulation pulse.

Statistical analyses requiring multiple comparisons were first examined with a nonparametric one-way analysis of variance (Kruskal–Wallis) followed by nonparametric tests between specific values. We considered comparisons to be significantly different if *P*-values were <0.05.

## Additional information

**How to cite this article:** Laprell, L. *et al.* Optical control of NMDA receptors with a diffusible photoswitch. *Nat. Commun.* 6:8076 doi: 10.1038/ncomms9076 (2015).

## Supplementary Material

Supplementary Information Supplementary Figures 1-10, Supplementary Note 1, Supplementary Methods and Supplementary References
