# Supplementary material for: Optical control of NMDA receptors with a diffusible photoswitch
Source: Nat Commun. 2015 Aug 27;6:8076. doi: 10.1038/ncomms9076 (PMC4560805; doi:10.1038/ncomms9076)
Supplement: Supplementary Information — Supplementary Figures 1-10, Supplementary Note 1, Supplementary Methods and Supplementary References [file ncomms9076-s1.pdf]

Optical control of NMDA receptors with a diffusible photoswitch

Laura Laprell[#,1], Emilienne Repak[#,2,3], Vilius Franckevicius,[1] Felix Hartrampf,[1] Jan Terhag[4], Michael Hollmann[4], Martin Sumser,[1] Nelson Rebola,[2,3] David A. DiGregorio*[2,3] and Dirk Trauner*[1]

[1]Department of Chemistry and Pharmacology, Ludwig-Maximilians-Universität, München, and Center for Integrated Protein Science, 81377 Munich, Germany.

[2]Institut Pasteur, Unit of Dynamic Neuronal Imaging, 25, rue du Dr Roux 75724 Paris Cedex 15, France

[3]CNRS UMR 3571, 2182, Genes, Synapses, and Cognition, Institut Pasteur, 25 rue du Dr. Roux, 75724 Paris Cedex 15, France

[4] Ruhr-Universität-Bochum, Department of Biochemistry, 44780 Bochum, Germany

[#]Authors contributed equally

*Corresponding authors

E-mail: david.digregorio@pasteur.fr, dirk.trauner@lmu.de

Table of contents

Supplementary figures S2

Supplementary note 1 ....S21

Supplementary methods S24

Experimental procedures S25

Supplementary references S30

Supplementary figures

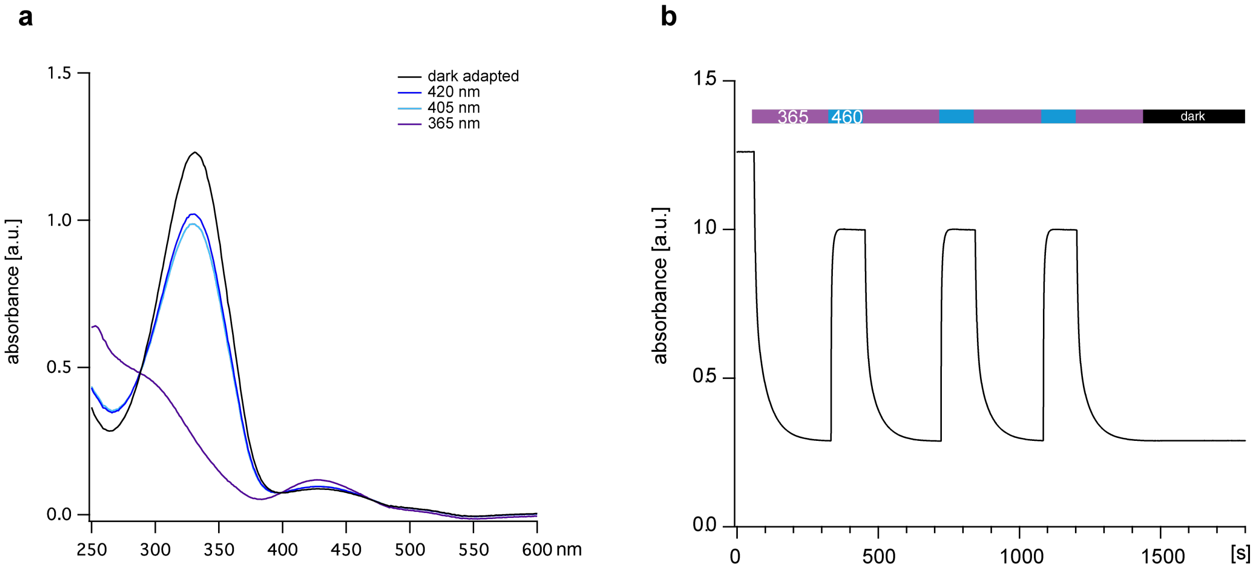

Supplementary Figure 1. UV-Vis spectra and thermal stability of ATG.

(a) UV-Vis spectra of ATG in the dark-adapted state and during illumination with 365, 405 and 420 nm light in physiological buffer. (b) Kinetics of the conversion of *trans*- to *cis*-ATG during illumination with 460 and 365 nm light (high power LEDs) measured at the maximal absorption wavelength of *trans*-ATG (330 nm).

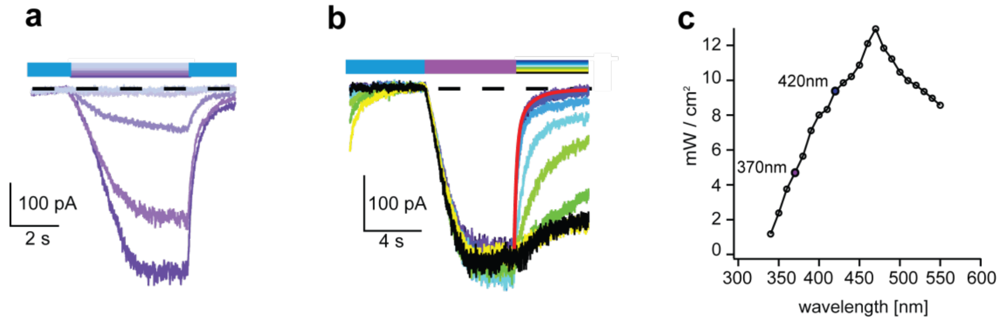

Supplementary Figure 2. Action spectrum, kinetics and dose-response curve recorded in layer 2/3 cortical neurons.

(a) Wavelength screening for activation of **ATG**-mediated (200 μM) currents. Raw data traces for 5 s light stimulation from 360 nm to 410 nm light (dark to light purple). (b) Wavelength screening for τ_{off} kinetics of **ATG**-mediated currents. Raw data traces showing the off-kinetics after a 5 s light-stimulation with 370 nm. Best τ_{off} kinetics were achieved at 425-450 nm light (red trace: exponential fit). (c) Relative light intensities depending on wavelengths used. Light intensity for *trans-cis* isomerization at 370 nm was 5 mW cm^{-2} and for *cis-trans* isomerization for 420 nm was 9.5 mW cm^{-2}.

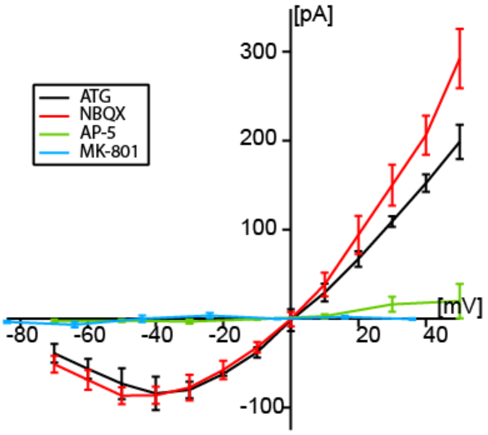

Supplementary Figure 3. ATG targets NMDARs

Current-voltage relationship of **ATG**-mediated currents measured after 3 s of illumination (370 nm) in the presence of NMDAR and AMPAR antagonists in layer 2/3 cortical neurons of acute brain slices. 200 µM **ATG** was used for all experiments. Black: control (n = 4_cells) Red: 25 µM NBQX (n = 8 cells), a selective AMPAR antagonist. Green: 40 µM D-AP-5, a selective NMDAR antagonist (n = 3 cells). Blue: 50 µM MK-801, a selective NMDAR blocker (n = 5 cells). Single data points represent mean ± SEM.

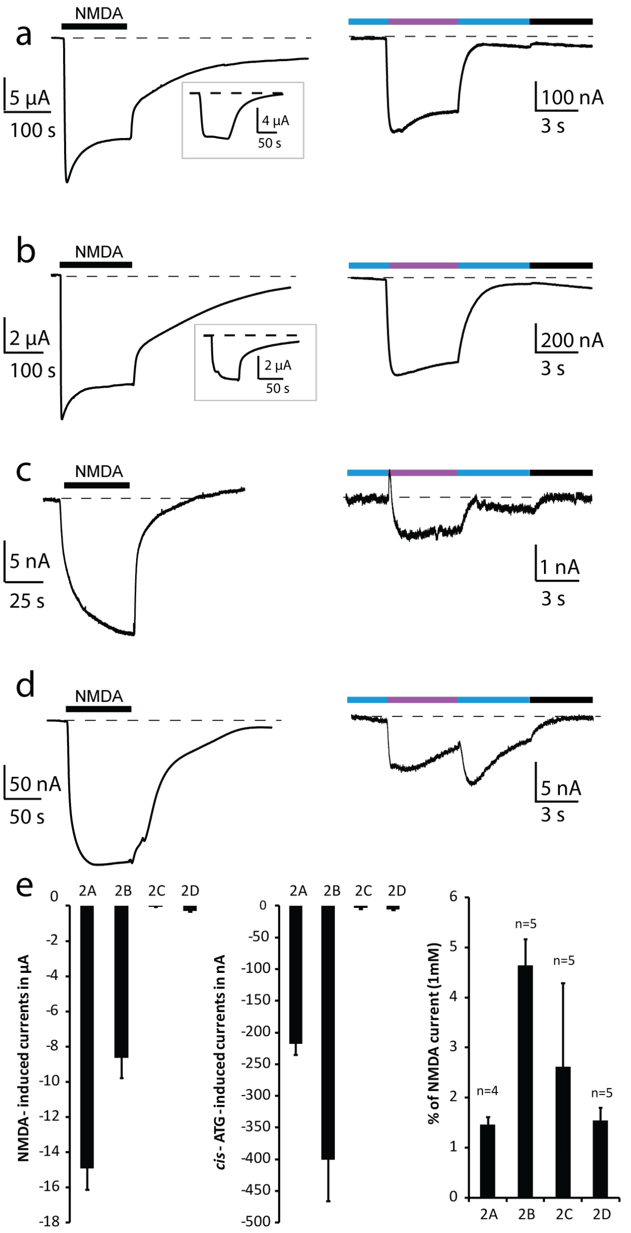

Supplementary Figure 4. ATG activates different NMDA receptor compositions in *Xenopus* oocytes.

NMDA- and *cis*-**ATG**-mediated currents in oocytes heterologously expressing NMDA receptor subunit combinations in the presence of 1.8 mM barium, 250 µM NFA and 10 µM glycine. Representative current traces in response to transient bath perfusion of 1 mM NMDA (left) and light-mediated currents (365 nm LED illumination, purple) in presence of 200 µM **ATG** (right) for the NMDA receptor combinations GluN1-1a and (a) GluN2A, (b) GluN2B, (c) GluN2C, and (d) GluN2D. Blue bar indicates 460 nm LED illumination. The insets in (a) and (b) show currents in response to non-saturating NMDA concentrations (10 µM), ruling out the presence of a calcium-induced chloride currents. 460 nm-evoked currents were present for

GluN2C and 2D containing receptors. (e) Summary plots showing average steady-state current amplitudes induced by 1 mM NMDA (left) and by 200 μM *cis*-**ATG** (center) for all subtype combinations shown in (a). Summary plot of the average within-cell ratio of *cis*-**ATG**-mediated currents in relation to saturating NMDA-induced currents (right). Average NMDA-evoked currents were larger (GluN1-1a/2A = −14.93±1.21 μA, GluN1-1a/2B = −8.65±1.13 μA, GluN1-1a/2C = −0.06±0.01 μA and GluN1-1a/2D = −0.29±0.05 μA) than *cis*-**ATG-**mediated currents (GluN1-1a/2A = −217.6±17.5 nA, GluN1-1a/2B = −400.9±65.4 nA, GluN1-1a/2C = −3.46±1.69 nA and GluN1-1a/2D = −5.91±0.57 nA).

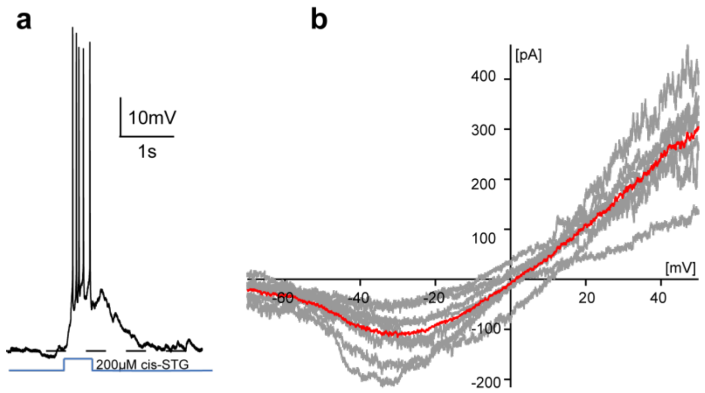

Supplementary Figure 5. Characterization of *cis*-STG in layer 2/3 cortical neurons. (a) Current-clamp recording in layer 2/3 cortical neuron with *cis*-**STG**. Puff-application of 200 µM *cis*-**STG** for 500 ms results in robust action potential firing. (b) Current-voltage relationship of *cis*-**STG** in cortical neurons. Ramps were performed between –70 mV and 50 mV. Single cell recordings are depicted in grey (n = 5 cells), average trace of all experiments is shown in red.

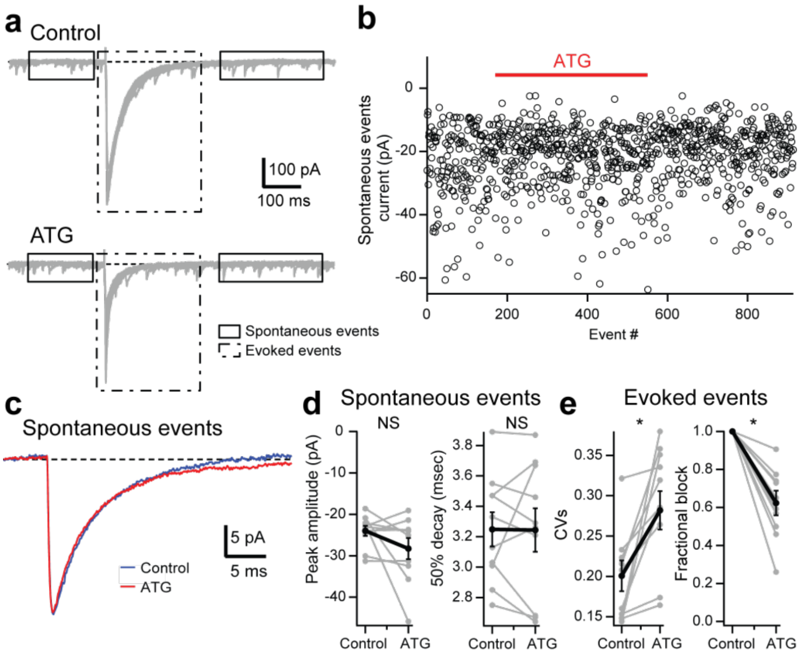

Supplementary Figure 6. Impact of ATG on GABA$_A$R-mediated currents. (a) Spontaneous and evoked IPSCs (elicited with 0.1 Hz stimulation) recorded from a CA1 pyramidal cell from an acute brain slice. (b) Peak amplitude of spontaneous events. After 10 minutes of recording, **ATG** (400 μM) was washed in for 20 minutes, and then subsequently washed out. (c) Average spontaneous IPSCs from example cell shown in a) in control conditions and in the presence of 400 μM **ATG**. (d) Summary plots showing the time at which spontaneous IPSCs decayed by 50% in control and in 400 μM **ATG** (n = 10 cells). (e) Summary plots showing the coefficient of variation and fractional block of IPSCs by 400 μM **ATG** (n = 10 cells). *p<0.05, NS indicates comparisons that are not significantly different (Wilcoxon matched pairs signed rank test).

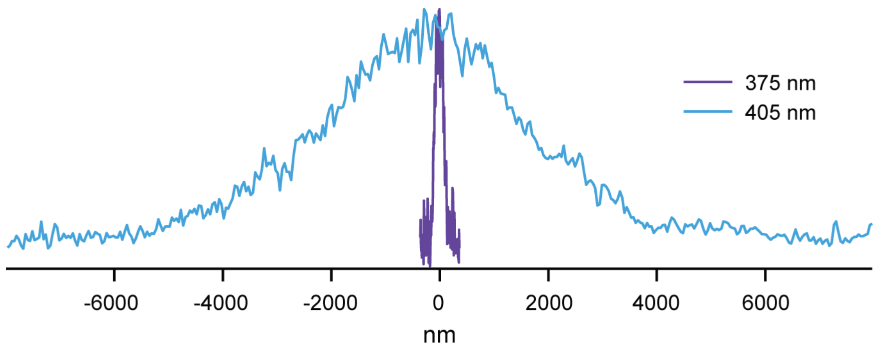

Supplementary Figure 7. One-photon 375 nm and 405 nm laser spot sizes. Relative intensity profiles resulting from scanning 100 nm green fluorescence beads with 375 nm (FWHM = 300 nm) or 405 nm (FWHM = 4.25 μm) laser illumination.

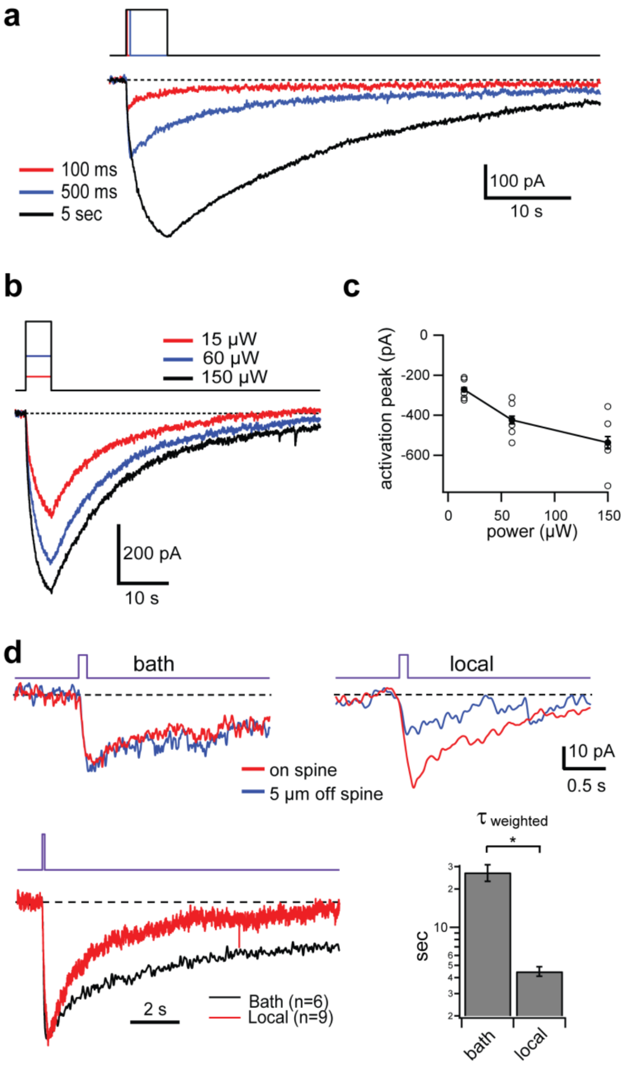

Supplementary Figure 8. NMDAR currents evoked in hippocampal CA1 pyramidal neurons in acute brain slices by one-photon activation of bath-applied ATG. (a) Representative *cis*-**ATG**-mediated currents in response to 100 ms, 500 ms, and 5 s 375 nm laser illumination of dendrites in the presence of 200 μM ATG and 5 μM NBQX. (b) *cis*-**ATG**-mediated currents were evoked with 5 s 375 nm laser pulses of 15 μW, 60 μW, and 150 μW. (c) The dependence of *cis*-**ATG**-mediated peak response on laser power (n = 5 cells). Increasing the power from 50 to 150 μW produces a sublinear increase in *cis*-**ATG**-mediated current amplitudes, suggesting responses are maximal, potentially due to saturation of **ATG** conversion or NMDAR occupancy. (d) Top: Distance dependence of *cis*-**ATG**-mediated responses evoked by 100 ms, 375 nm light pulses using bath and local **ATG** application (100 μM). A more localized response is achieved using local application of **ATG**. Traces are averages of 3 trials. Bottom: Decay kinetics of *cis*-**ATG**-mediated responses (100 ms illumination) in bath and local application. Summary bar graph shows weighted decay time constants ($\tau_{weighted}$) for bath (27.1 ± 4.0 s, n = 6 cells) and local perfusion (local, 4.5 ± 0.4 s, n = 9 cells). *p<0.05 (Wilcoxon matched pairs signed rank test).

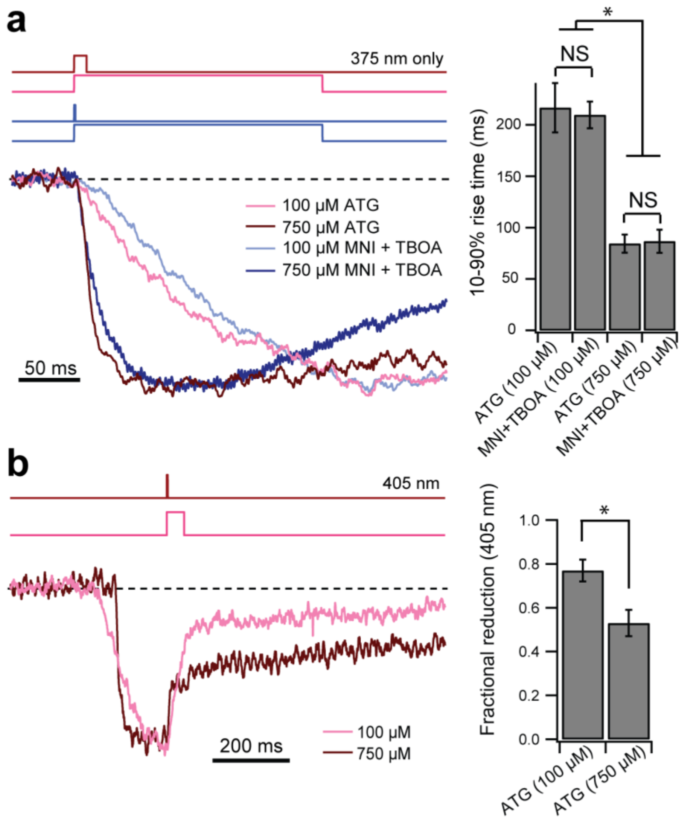

Supplementary Figure 9. Concentration dependence of activation and fractional reduction of CA1 pyramidal cell NMDAR currents for ATG and MNI-glutamate. (a) *cis*-**ATG**-mediated currents and MNI-glutamate uncaging-evoked currents (**ATG**: 100 µM (n = 9 cells), and 750 µM (n = 12 cells); MNI-glutamate: 100 µM (n = 5 cells), 750 µM (n = 5 cells)). **ATG** and MNI-glutamate were locally applied. MNI-glutamate experiments were performed in TBOA (500 µM). There is no significant difference between rise times of NMDAR currents evoked by MNI-glutamate uncaging and **ATG** activation for the same concentration. (b) Comparison of 405 nm-mediated reduction in NMDAR currents following 375 nm activation at different concentrations: 77 ± 5% for 100 µM (n = 8 cells) and 53 ± 6% for 750 µM (n = 6 cells). Fractional reduction was calculated as $1 - \frac{50\ ms\ average\ immediately\ after\ 405\ nm\ pulse}{50\ ms\ average\ immediately\ before\ 405\ nm\ pulse}$. *p<0.05 (Steel Dwass all pairs nonparametric multiple comparison test). The fractional reduction in *cis*-**ATG**-mediated currents by 405 nm light was most effective when working at the lower **ATG** concentration, 100 µM.

a

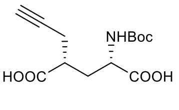
NHBoc
HOOC
COOH

1

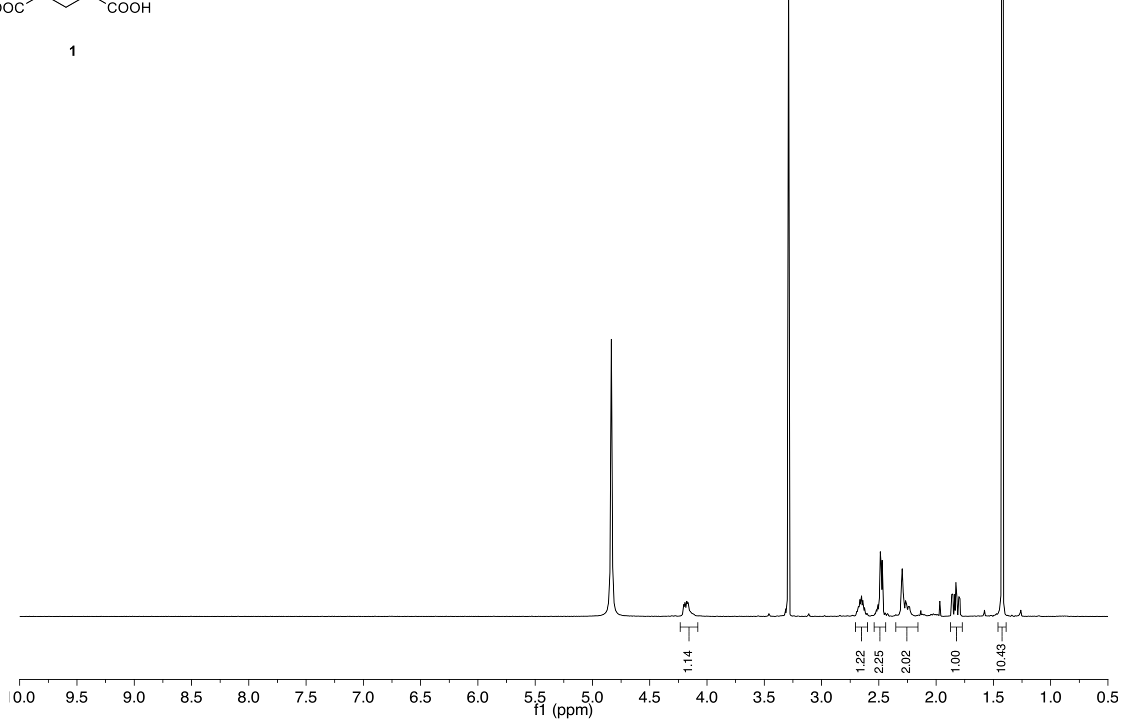
1.14
1.22
2.25
2.02
1.00
10.43
10.0 9.5 9.0 8.5 8.0 7.5 7.0 6.5 6.0 5.5 5.0 4.5 4.0 3.5 3.0 2.5 2.0 1.5 1.0 0.5
f1 (ppm)

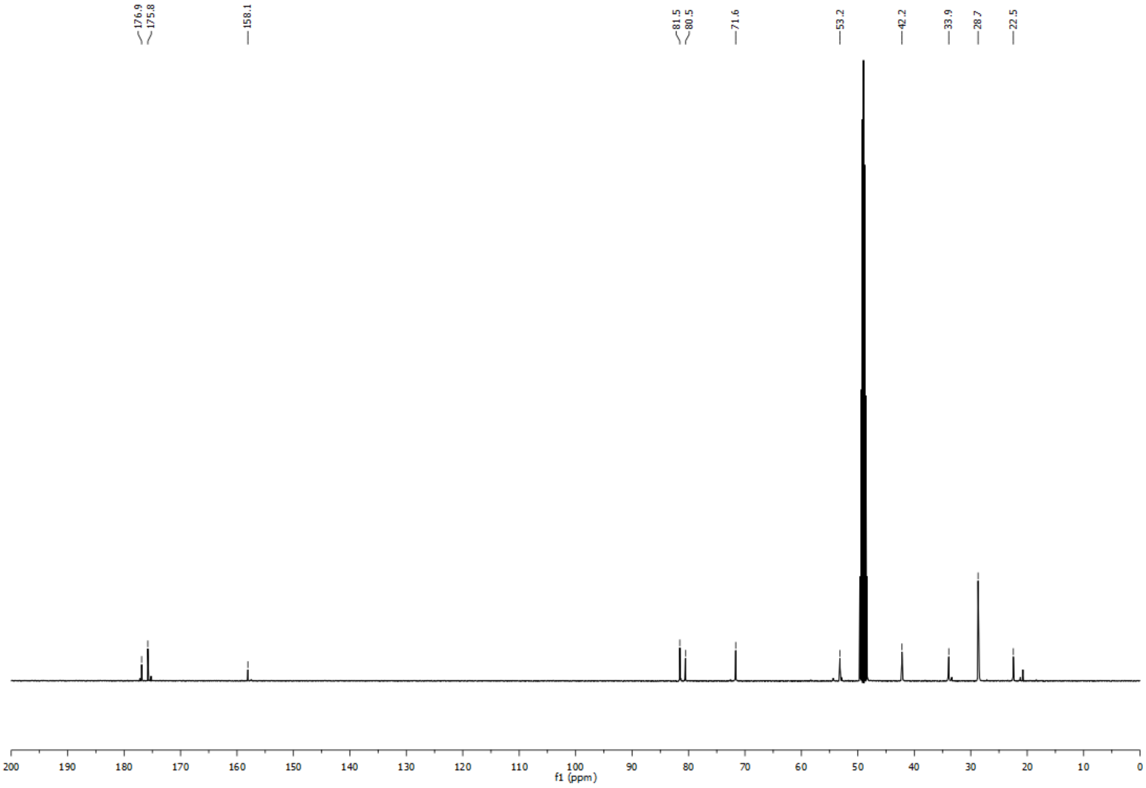
176.9
175.8
158.1
81.5
80.5
71.6
53.2
42.2
33.9
28.7
22.5
200 190 180 170 160 150 140 130 120 110 100 90 80 70 60 50 40 30 20 10 0
f1 (ppm)

b

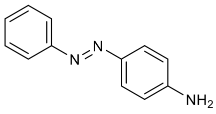
N
N
NH2

S3

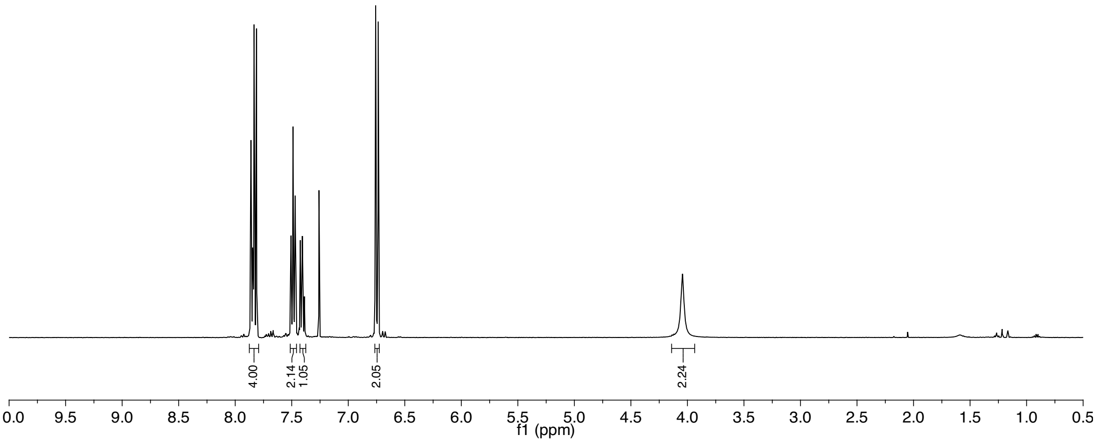
4.00
2.14
1.05
2.05
2.24
f1 (ppm)

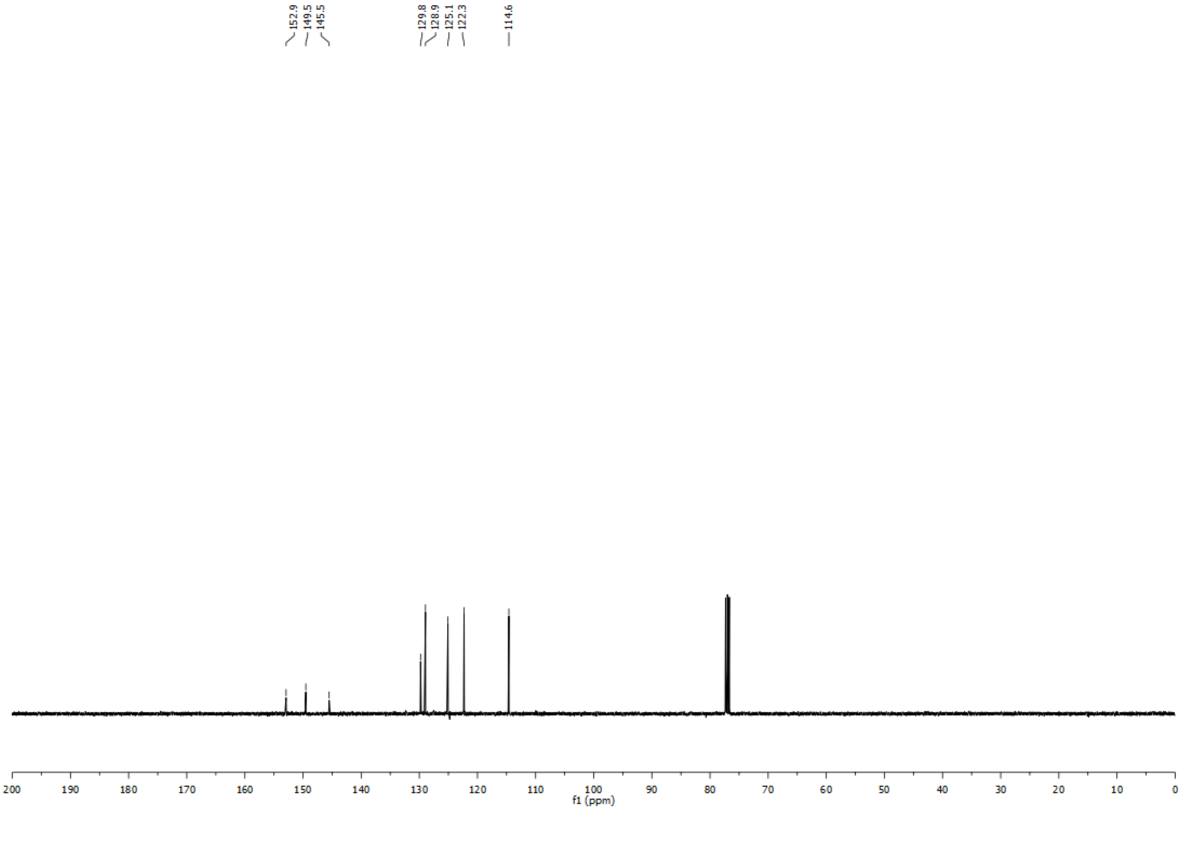
152.9
149.5
145.5
129.8
128.9
125.1
122.3
114.6
f1 (ppm)

c

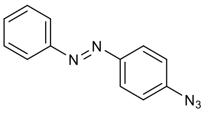
N
N
N3

2

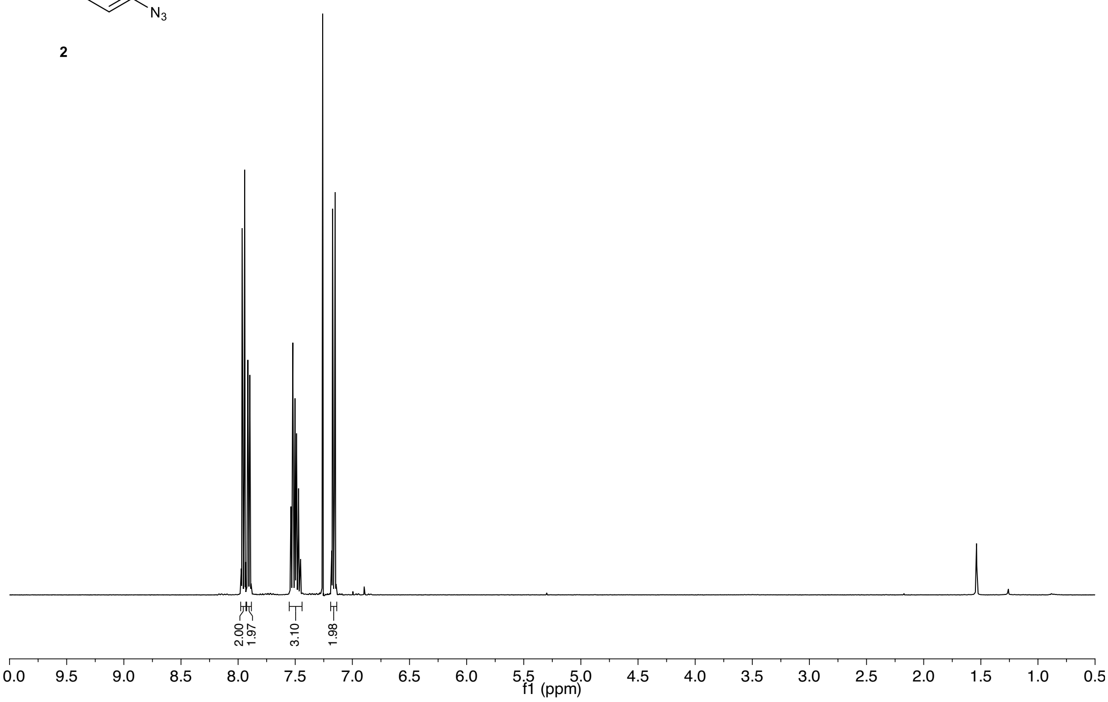
2.00
1.97
3.10
1.98
0.0 9.5 9.0 8.5 8.0 7.5 7.0 6.5 6.0 5.5 5.0 4.5 4.0 3.5 3.0 2.5 2.0 1.5 1.0 0.5
f1 (ppm)

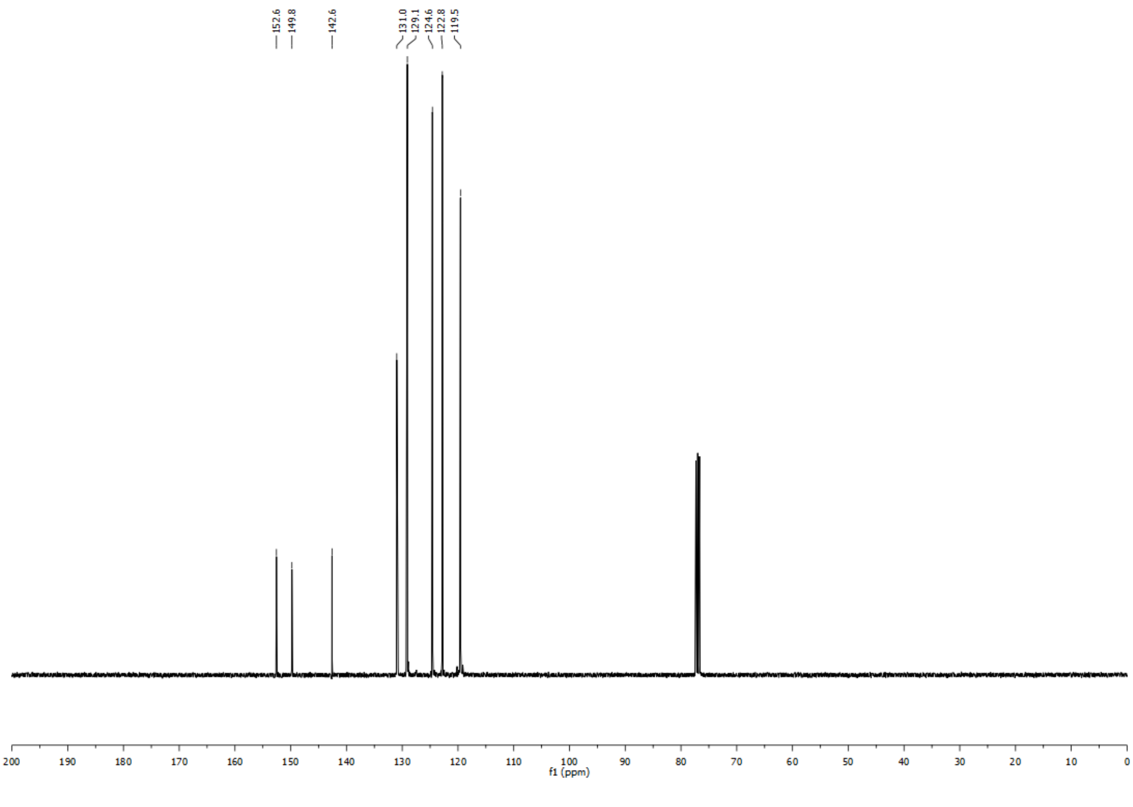
152.6
149.8
142.6
131.0
129.1
124.6
122.8
119.5
200 190 180 170 160 150 140 130 120 110 100 90 80 70 60 50 40 30 20 10 0
f1 (ppm)

d

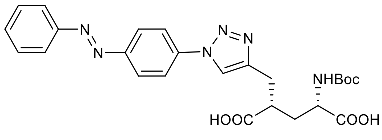
N
N
N
N
N
NHBoc
HOOC
COOH

3

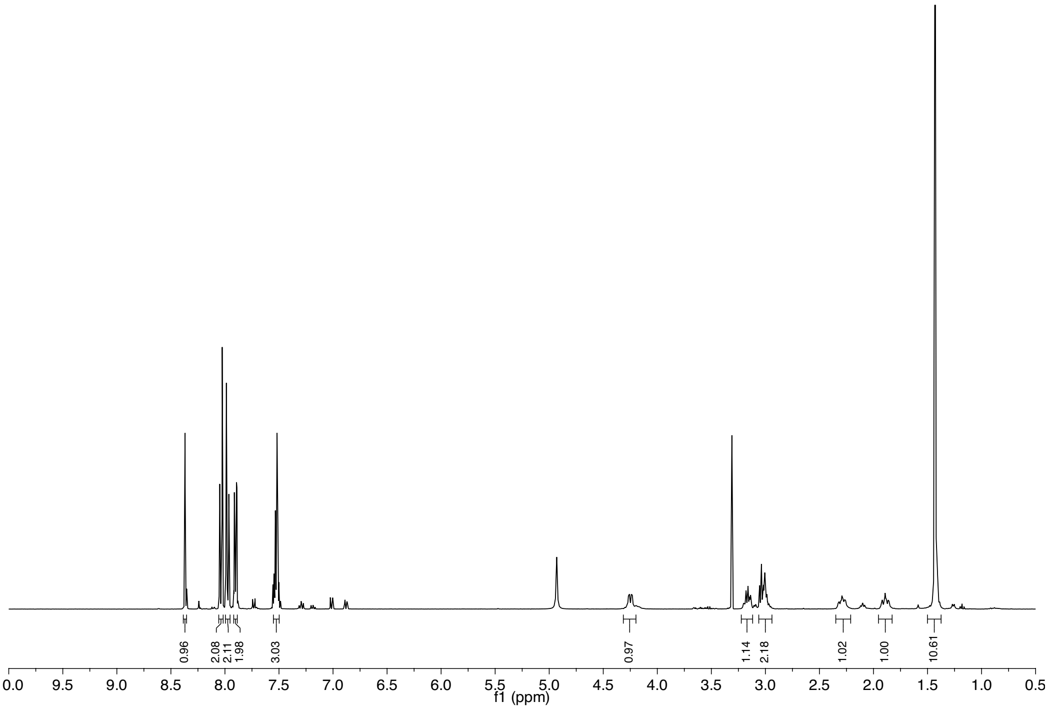
0.96
2.08
2.11
1.98
3.03
0.97
1.14
2.18
1.02
1.00
10.61
0.0 9.5 9.0 8.5 8.0 7.5 7.0 6.5 6.0 5.5 5.0 4.5 4.0 3.5 3.0 2.5 2.0 1.5 1.0 0.5
f1 (ppm)

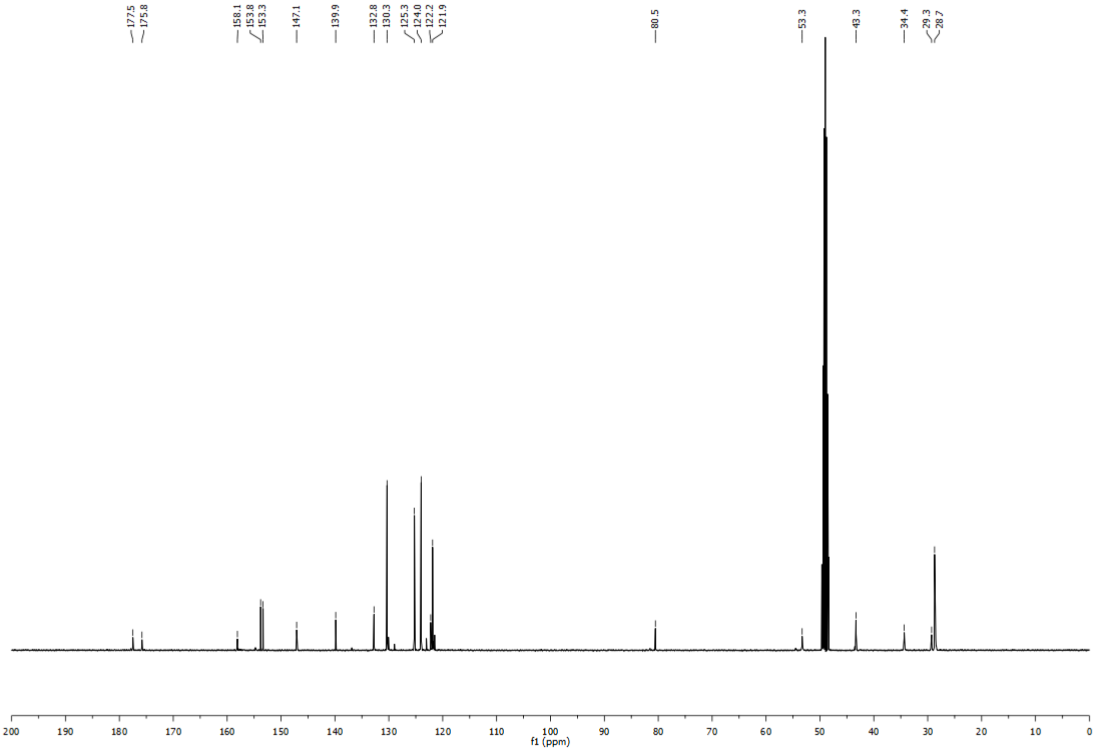
177.5
175.8
158.1
153.8
153.3
147.1
139.9
132.8
130.3
125.3
124.0
122.2
121.9
80.5
53.3
43.3
34.4
29.3
28.7
200 190 180 170 160 150 140 130 120 110 100 90 80 70 60 50 40 30 20 10 0
f1 (ppm)

e

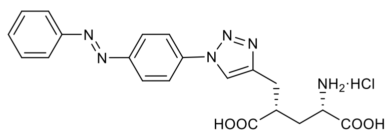
N
N
N
N
N
N
NH2·HCl
HOOC
COOH

trans-**ATG**

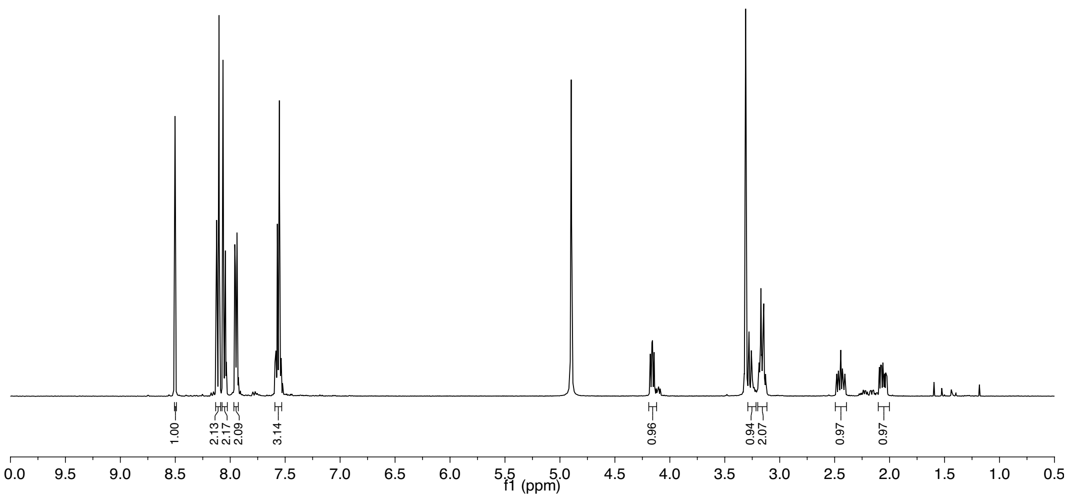
1.00
2.13
2.17
2.09
3.14
0.96
0.94
2.07
0.97
0.97
0.0
9.5
9.0
8.5
8.0
7.5
7.0
6.5
6.0
5.5
5.0
4.5
4.0
3.5
3.0
2.5
2.0
1.5
1.0
0.5
f1 (ppm)

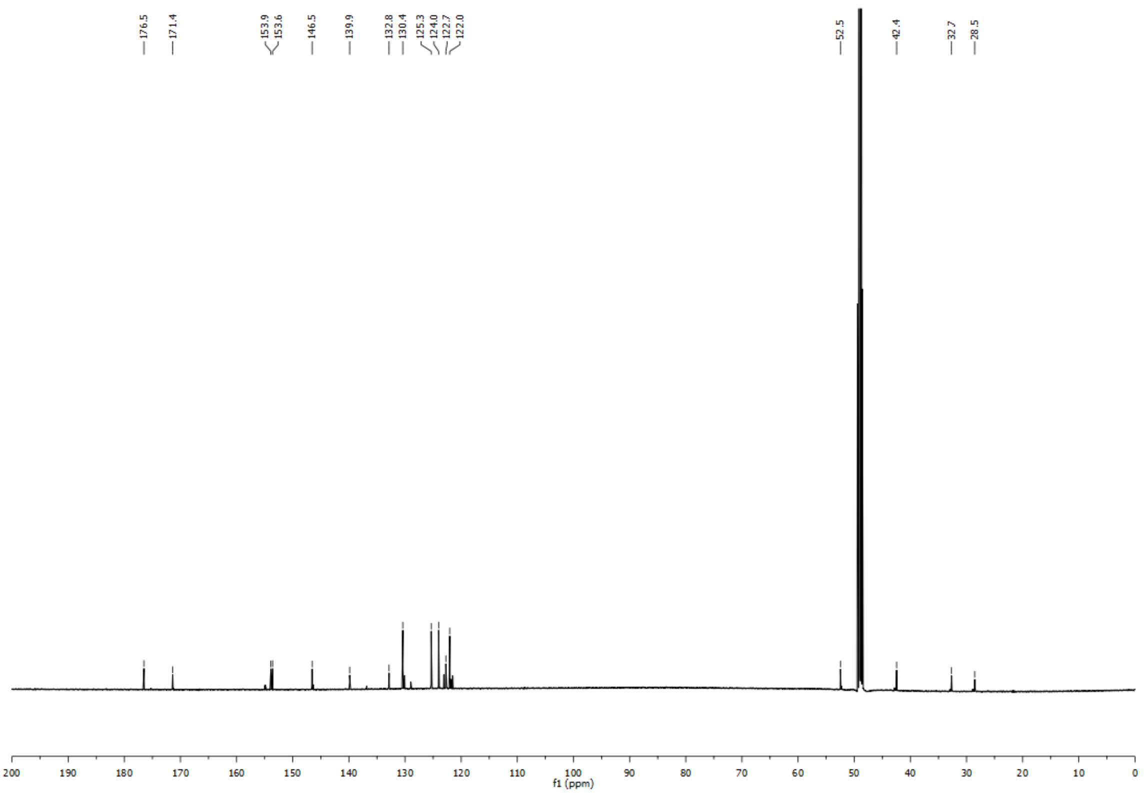
176.5
171.4
153.9
153.6
146.5
139.9
132.8
130.4
125.3
124.0
122.7
122.0
52.5
42.4
32.7
28.5
200
190
180
170
160
150
140
130
120
110
100
90
80
70
60
50
40
30
20
10
0
f1 (ppm)

f

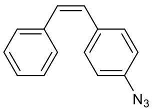

4

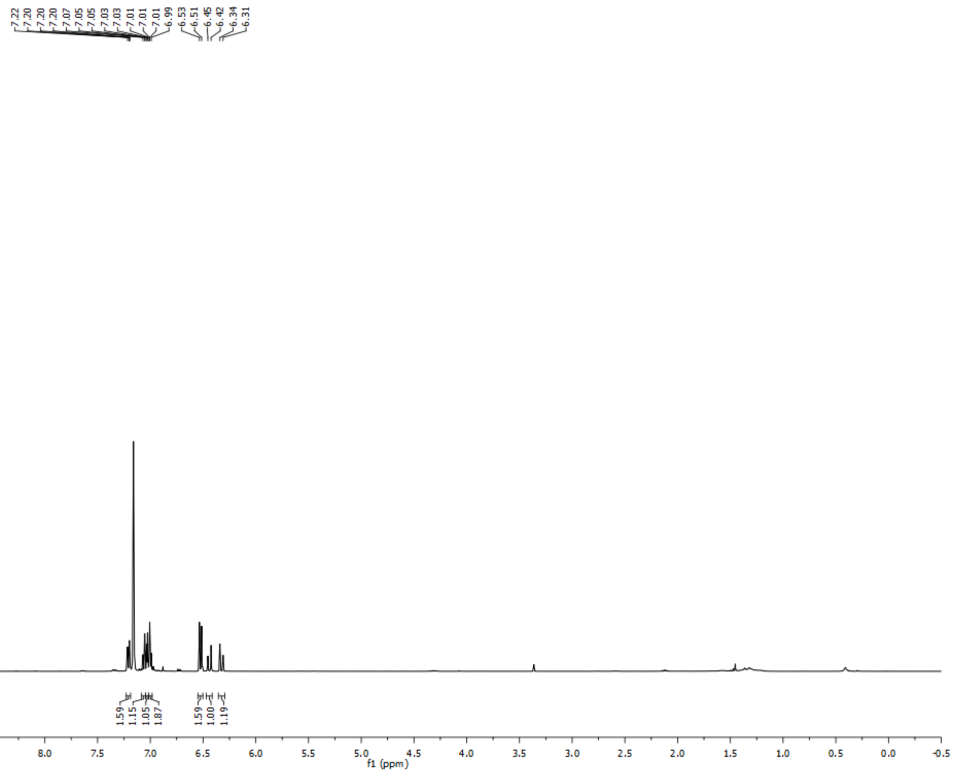
7.22
7.20
7.20
7.20
7.07
7.05
7.05
7.03
7.03
7.01
7.01
7.01
6.99
6.53
6.51
6.45
6.42
6.34
6.31
1.59
1.15
1.05
1.87
1.59
1.00
1.19
10.0 9.5 9.0 8.5 8.0 7.5 7.0 6.5 6.0 5.5 5.0 4.5 4.0 3.5 3.0 2.5 2.0 1.5 1.0 0.5 0.0 -0.5
f1 (ppm)

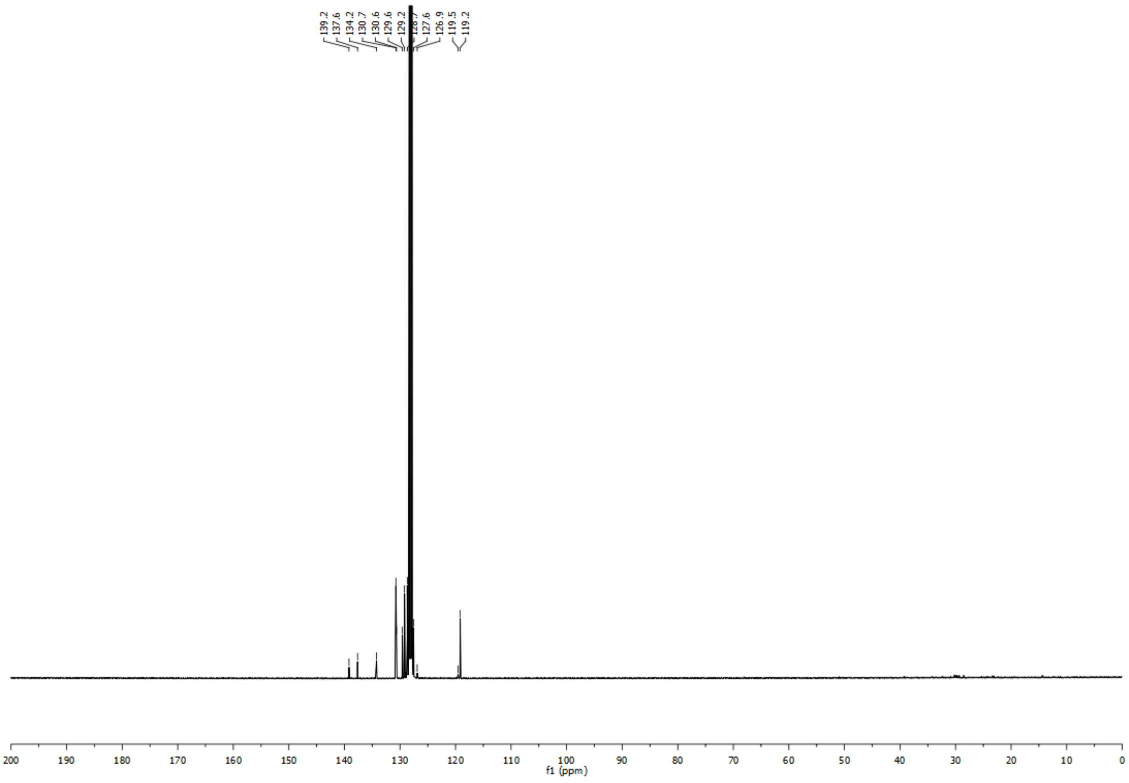
139.2
137.6
134.2
130.7
130.6
129.6
129.2
128.7
127.6
126.9
119.5
119.2
200 190 180 170 160 150 140 130 120 110 100 90 80 70 60 50 40 30 20 10 0
f1 (ppm)

g

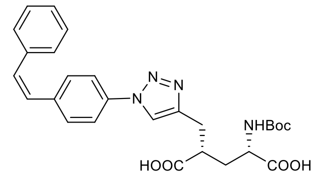

5

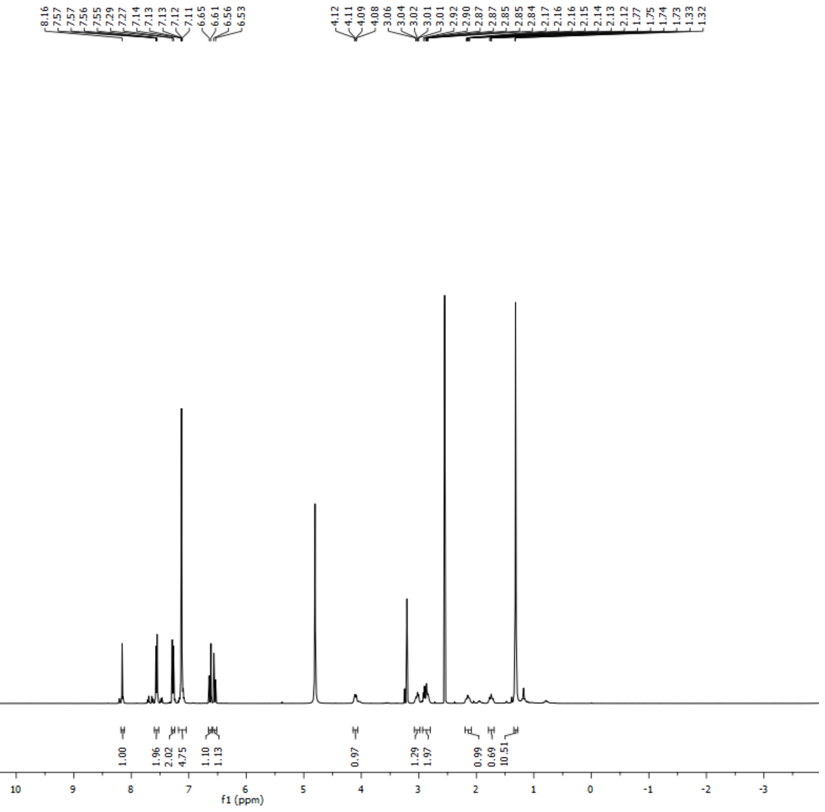

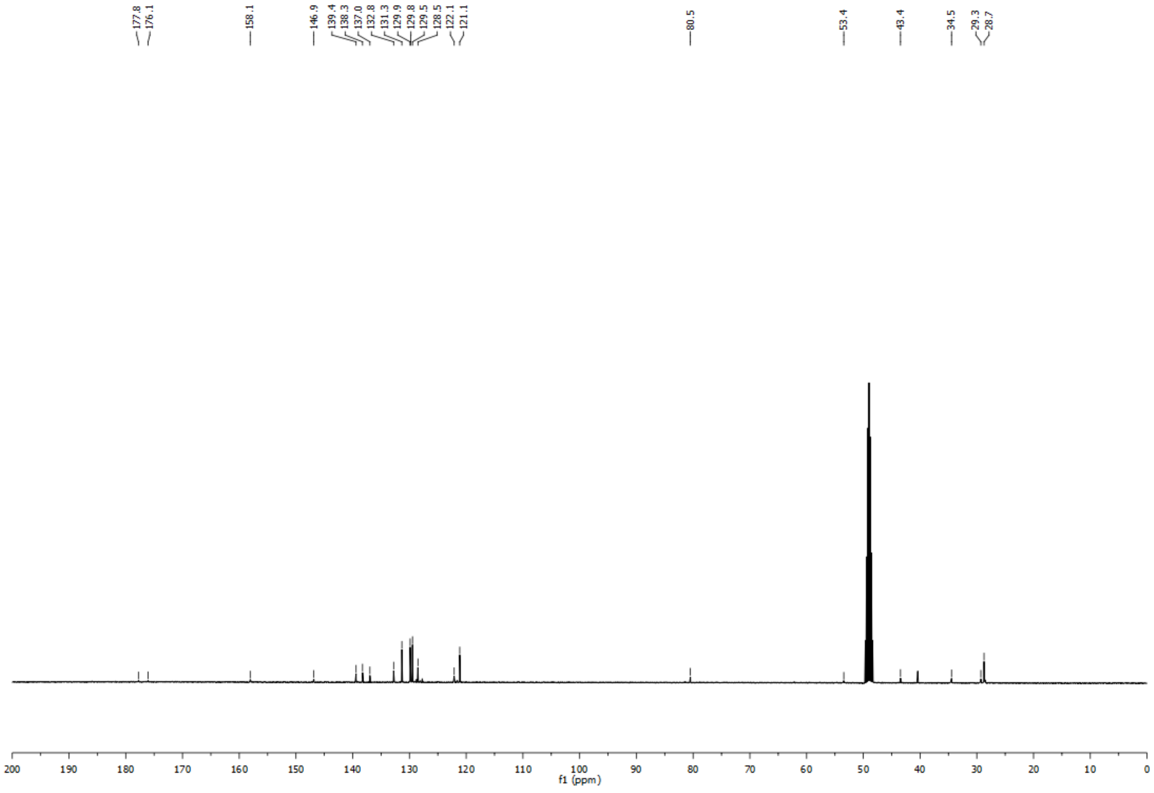

h

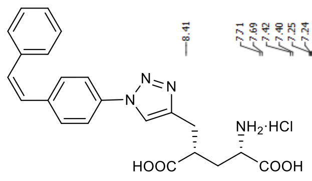

cis-**STG**

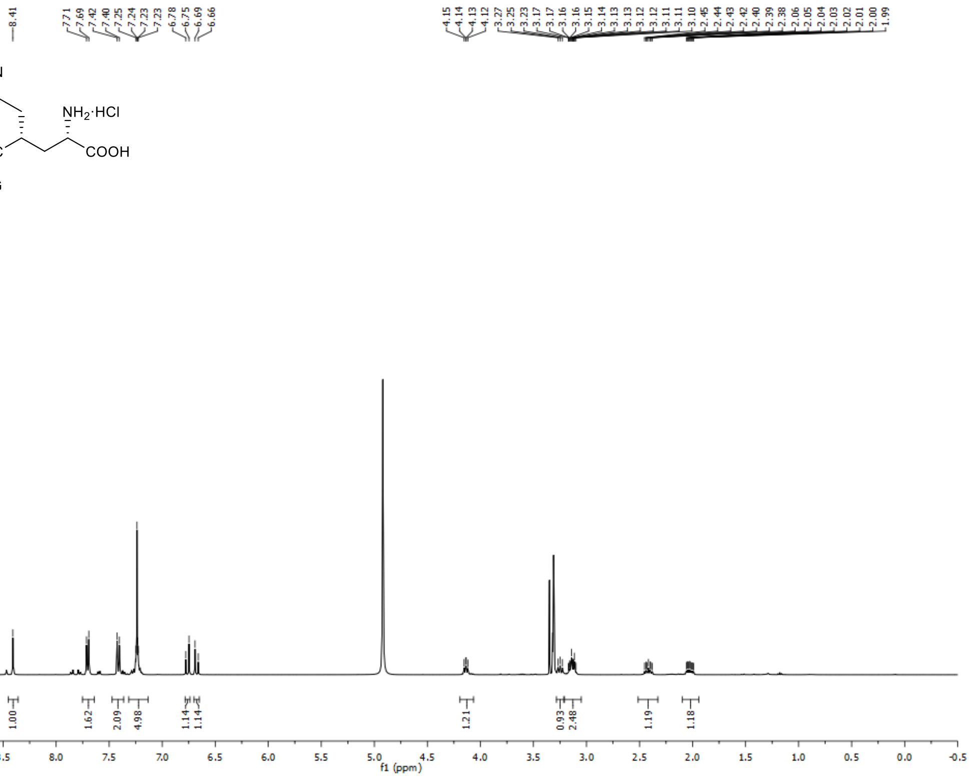

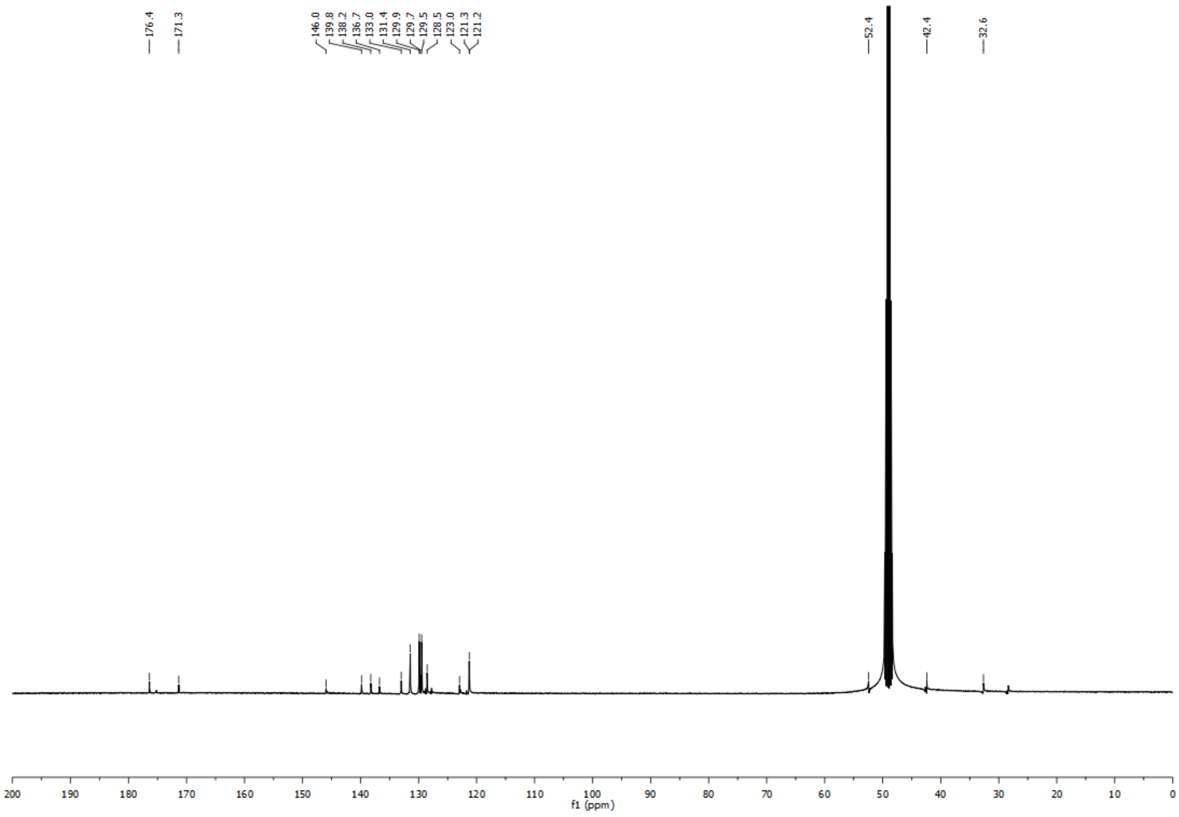

Supplementary Figure 10 ^{1}H- (top) and ^{13}C-NMR (bottom) spectra of intermediates and final products.

(a) NMR spectra of compound **1**. (b) NMR spectra of compound **S3**. (c) NMR spectra of compound **2**. (d) NMR spectra of compound **3**. (e) NMR spectra of *trans*-**ATG**. (f) NMR spectra of compound **4**. (g) NMR spectra of compound **5**. (h) NMR spectra of compound *cis*-**STG**.

Supplementary note 1

All reactions were performed with standard Schlenk techniques under an atmosphere of nitrogen or argon in oven-dried glassware (100 °C oven temperature) unless specified otherwise. Tetrahydrofuran (THF) and diethyl ether (Et_2O) were distilled prior to use from sodium and benzophenone, triethylamine (Et_3N), diethylisopropylamine (DIPEA) and dichloromethane (CH_2Cl_2) were distilled from calcium hydride. *N*,*N*-dimethylformamide (DMF), acetonitrile (MeCN), toluene and methanol (MeOH) were purchased from Acros Organics as 'extra dry' reagents under inert gas atmosphere and over molecular sieves. All other reagents were purchased from commercial sources and used without further purification.

Reaction progress was monitored by analytical thin-layer chromatography (TLC), which was carried out using pre-coated glass plates (silica gel 60 F254) from Merck. Visualization was achieved by exposure to ultraviolet light (UV, 254 nm) where applicable followed by staining with aqueous acidic ceric ammonium molybdate(IV) (CAM) or potassium permanganate solution. Flash column chromatography was performed using Merck silica gel (40–63 µm particle size). For reversed phase (RP) TLC, pre-coated glass plates (silica gel C18 RP-18W/UV254) from Macherey-Nagel were used, and preparative RP columns were performed on Waters silica gel (Preparative C18, 125 Å, 55–105 µm).

Proton nuclear magnetic resonance (^1H NMR) spectra were recorded on a Varian 300, Varian 400, Inova 400 or Varian 600 spectrometer. Chemical shifts (δ scale) are expressed in parts per million (ppm) and are calibrated using residual protic solvent as an internal reference ($CHCl_3$: δ = 7.26 ppm, MeOH-d_3: δ = 3.31 ppm). Data for ^1H NMR spectra are reported as follows: chemical shift (δ ppm) (multiplicity, coupling constants (Hz), integration). Couplings are expressed as: s = singlet, d = doublet, t = triplet, q = quartet, m = multiplet, br = broad, or combinations thereof. Carbon nuclear magnetic resonance (^{13}C NMR) spectra were recorded on the same spectrometers at 75, 100 and 150 MHz (±1 MHz variance). Carbon chemical shifts (δ scale) are also expressed in parts per million (ppm) and are referenced to the central carbon resonances of the solvents ($CDCl_3$: δ = 77.16 ppm, MeOH-d_4: δ = 49.00 ppm). In order to assign the ^1H and ^{13}C NMR spectra, a range of 2D NMR experiments (COSY, HMQC, HMBC, NOESY) was used as appropriate.

Infrared (IR) spectra were recorded on a Perkin Elmer Spectrum BX II (FTIR System) equipped with an attenuated total reflection (ATR) measuring unit. IR data is reported in frequency of absorption (cm^{-1}). The IR bands are characterized as: w = weak, m = medium, s = strong, br = broad or combinations thereof.

Mass spectroscopy (MS) experiments were performed on a Thermo Finnigan MAT 95 (electron ionization, EI) or on a Thermo Finnigan LTQ FT (electrospray ionization, ESI) instrument.

Optical rotations were measured at 22 °C on a Perkin-Elmer 241 polarimeter using a sodium lamp (*l* = 589 nm, D-line) within a cell with a path length (*l*) of 0.5 dm. Concentrations (*c*) are expressed in g/(100 mL). Specific rotations were calculated using the equation $[\alpha]^{24} = 100 \cdot \alpha/(c \cdot l)$ and are reported in 10^{-1}deg cm^2g^{-1}.

Supplementary methods

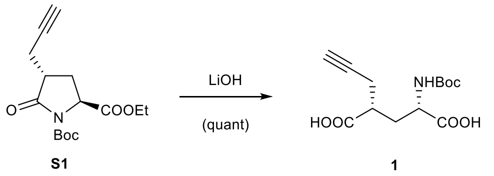

for **ATG**

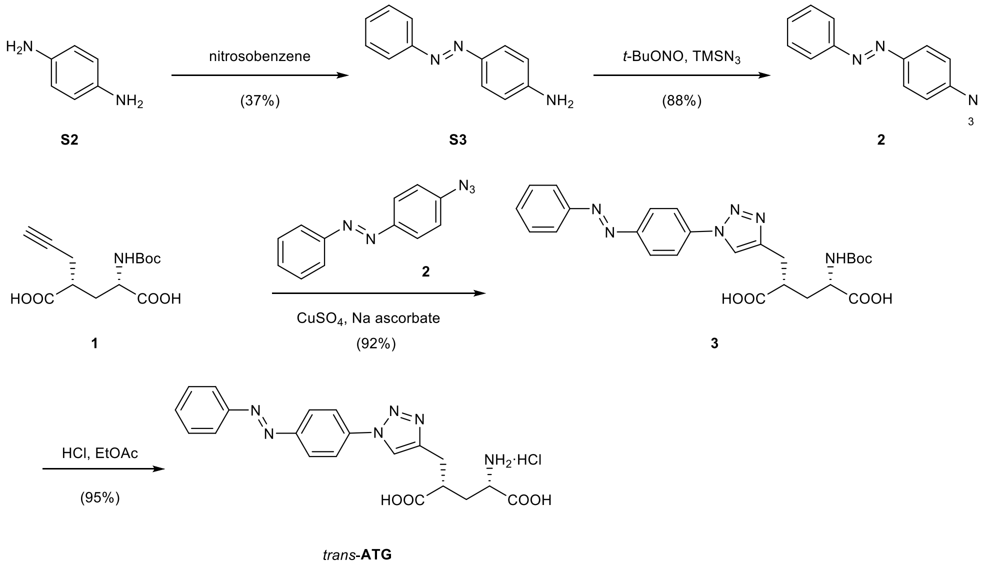

for *cis*-**STG**

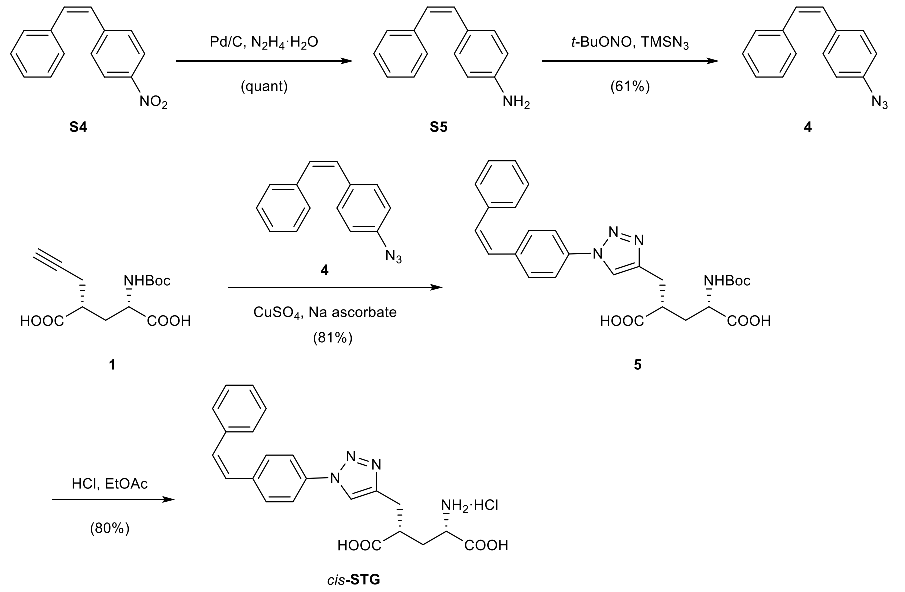

Experimental procedures

Propargylated *N*-Boc-L-pyroglutamic acid ethyl ester (**S1**) was synthesized following a literature procedure.[1]

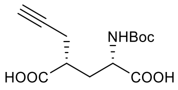

1

(2*S*,4*R*)-2-(*tert*-Butoxycarbonylamino)-4-(prop-2-ynyl)pentanedioic acid (1). To a solution of propargylated pyroglutamate **S1** (528 mg, 1.79 mmol) in THF (50 mL) cooled to 0 ºC was added aq. LiOH (1 M, 50 mL). The resulting mixture was allowed to warm to room temperature and stirred at this temperature for 30 min. The solution was acidified to pH=1 with aq. HCl (1 N, 100 mL) and extracted with EtOAc (2 x 200 mL). The combined organic phase was washed with brine (400 mL), then dried ($MgSO_4$) and concentrated *in vacuo*. Flash column chromatography (CH_2Cl_2:MeOH:AcOH:H_2O 90:10:0.6:0.6) afforded diacid **1** (510 mg, quant.) as a white foam.

R_f = 0.23 (CH_2Cl_2:MeOH:AcOH:H_2O 90:10:0.6:0.6); $[\alpha]_D^{22}$ –12.0 (*c* 3.1, MeOH); mp 47-49 °C; IR (ATR): 3296, 2979, 2934, 2556 (br), 1706, 1515 cm^{-1}; δ_H (400 MHz, CD_3OD) 4.21 (dd, *J* = 10.6, 4.2 Hz, 1H), 2.72 – 2.63 (m, 1H), 2.55 – 2.48 (m, 2H), 2.35 – 2.31 (m, 1H), 2.31 – 2.24 (m, 1H), 1.84 (ddd, *J* = 14.2, 10.7, 4.0 Hz, 1H), 1.44 (s, 9H); δ_C (100 MHz, CD_3OD) 176.9, 175.8, 158.1, 81.5, 80.5, 71.6, 53.2, 42.2, 33.9, 28.7, 22.5; HRMS (ESI): calculated for $C_{13}H_{19}NO_6^-$: 284.1140, found: 284.1147 $[M–H]^-$.

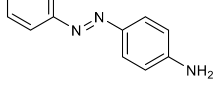

S3

(*E*)-4-(Phenyldiazenyl)aniline (S3).[2] To a solution of nitrosobenzene (749 mg, 7.0 mmol) in acetic acid (50 mL) was added 1,4-phenylenediamine (**S2**) (756 mg, 7.0 mmol) as a solid and the resulting mixture was stirred at 40 °C for 15 h. The dark solution was allowed to cool to room temperature, then diluted with water (300 mL) and extracted with EtOAc (3 x 300 mL). The combined organic phase was washed further with brine (2 x 500 mL), then dried ($MgSO_4$) and concentrated *in vacuo*. Flash column chromatography (dry-loaded, hexanes:EtOAc 4:1) afforded (*E*)-4-(phenyldiazenyl)aniline (**S3**) (510 mg, 37%) as an orange solid: R_f = 0.24 (hexanes:EtOAc 4:1); mp 116-119 °C; IR (ATR): 3348, 1616, 1596 cm^{-1}; δ_H (400 MHz, $CDCl_3$) 7.87 – 7.80 (m, 4H), 7.49 (t, *J* = 7.5 Hz, 2H), 7.41 (t, *J* = 7.4 Hz, 1H), 6.75

(d, J = 8.9 Hz, 2H), 4.04 (br s, 2H); δ_C (100 MHz, $CDCl_3$) 152.9, 149.5, 145.5, 129.8, 128.9, 125.1, 122.3, 114.6; HRMS (EI) calculated for $C_{12}H_{11}N_3^+$: 197.0947 M^+, found: 197.0954 M^+.

(*E*)-4-(Phenyldiazenyl)aniline can also be prepared via a modified literature procedure.[2] To a suspension of aniline **S2** (9.34 g, 100 mmol) in water (200 mL) was added solid sodium carbonate (5.30 g, 50 mmol) and the mixture was cooled to 0 ºC. A solution of sodium nitrite (7.40 g, 107 mmol) in water (100 mL), half-concentrated aq. HCl (40 mL), and a solution of aniline (9.34 g, 100 mL) in acetic acid (6 mL) were all slowly added in a sequential manner with careful monitoring of the temperature. The mixture was stirred at 0 ºC for 30 min, at which point aq. NaOH (12%, 100 mL) was added, resulting in the formation of an orange solid. The mixture was allowed to warm to room temperature and vigorously stirred at this temperature for 2 h. The suspension was diluted with brine (200 mL) and extracted with CH_2Cl_2 (500 mL). The organic phase was dried ($MgSO_4$) and concentrated *in vacuo*. Flash column chromatography (dry-loaded, hexanes:EtOAc 9:1-4:1) afforded (*E*)-4-(phenyldiazenyl)aniline (**S3**) (5.72 g, 30%) as an orange solid.

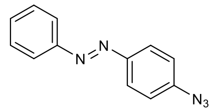

2

(*E*)-1-(4-Azidophenyl)-2-phenyldiazene (2).[3] Azide **2** was prepared by diazotization and displacement with azide using a literature procedure.[4] To a solution of (*E*)-4-(phenyldiazenyl)aniline (99 mg, 0.50 mmol) in MeCN at 0 °C was added *tert*-butyl nitrite (89 µL, 0.75 mmol) followed by trimethylsilyl azide (97%, 80 µL, 0.60 mmol). The mixture was allowed to warm to room temperature and stirred at this temperature for 1 h, then concentrated *in vacuo*. Flash column chromatography (dry-loaded, hexanes:CH_2Cl_2 9:1) afforded azide **2** (98 mg, 88%) as an orange solid.

R_f = 0.20 (hexanes:CH_2Cl_2 9:1); mp 90-91 °C; IR (ATR): 2107, 1592 cm^{-1}; δ_H (400 MHz, $CDCl_3$) 7.95 (d, J = 9.0 Hz, 2H), 7.91 (dd, J = 8.3, 1.5 Hz, 2H), 7.55 – 7.44 (m, 3H), 7.16 (d, J = 9.0 Hz, 2H); δ_C (100 MHz, $CDCl_3$) 152.6, 149.8, 142.6, 131.0, 129.1, 124.6, 122.8, 119.5; HRMS (EI): calculated for $C_{12}H_9N_5^+$: 223.0852 M^+, found: 223.0858 M^+.

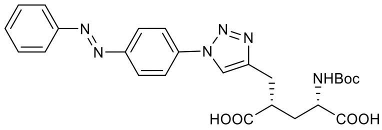

3

(2*S*,4*S*)-2-(*tert*-Butoxycarbonylamino)-4-((1-(4-((*E*)-phenyldiazenyl)phenyl)-1*H*-1,2,3-triazol-4-yl)methyl)pentanedioic acid (3). Alkyne **1** (215 mg, 0.75 mmol) and azide **2** (168 mg, 0.75 mmol) were dissolved in a mixture of MeOH (12 mL) and DMSO (4 mL) by means of ultrasound. Copper(II) sulfate pentahydrate (38 mg, 0.15 mmol), sodium ascorbate (37 mg, 0.19 mmol) and water (4 mL) were added, and the resulting suspension was stirred at room temperature for 24 h. The reaction was allowed to cool to room temperature, then quenched with aq. HCl (1 N, 50 mL) and extracted with EtOAc (3 x 50 mL). The combined organic phase was washed with aq. LiCl (10%, 3 x 100 mL) and brine (100 mL), dried ($MgSO_4$) and concentrated *in vacuo*. Flash column chromatography (CH_2Cl_2:MeOH:AcOH:H_2O 90:10:0.6:0.6) afforded the Boc-protected amine **3** (353 mg, 92%) as an orange solid.

R_f = 0.26 (CH_2Cl_2:MeOH:AcOH:H_2O 90:10:0.6:0.6); $[\alpha]_D^{22}$ +4.9 (*c* 1.63, MeOH); decomp. 136 °C; IR (ATR): 2978, 2930, 2508 (br), 1700, 1603, 1507 cm^{-1}; δ_H (400 MHz, CD_3OD, major *E* isomer quoted) 8.37 (s, 1H), 8.04 (d, *J* = 9.1 Hz, 2H), 7.98 (d, *J* = 9.1 Hz, 2H), 7.92 – 7.88 (m, 2H), 7.56 – 7.50 (m, 3H), 4.25 (dd, *J* = 10.6, 3.9 Hz, 1H), 3.21 – 3.12 (m, 1H), 3.07 – 2.93 (m, 2H), 2.29 (ddd, *J* = 13.5, 9.7, 3.9 Hz, 1H), 1.89 (ddd, *J* = 13.6, 10.7, 3.3 Hz, 1H), 1.43 (s, 9H); δ_C (100 MHz, CD_3OD, major *E* isomer quoted) 177.5, 175.8, 158.1, 153.8, 153.3, 147.1, 139.9, 132.8, 130.3, 125.3, 124.0, 122.2, 121.9, 80.5, 53.3, 43.3, 34.4, 29.3, 28.7; HRMS (ESI): calculated for $C_{25}H_{28}N_6O_6$: 509.2143 $[M+H]^+$, found: 509.2145 $[M+H]^+$.

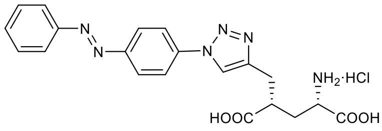

trans-**ATG**

(2*S*,4*S*)-2-Amino-4-((1-(4-((*E*)-phenyldiazenyl)phenyl)-1*H*-1,2,3-triazol-4-yl)methyl)pentanedioic acid hydrochloride (*trans*-ATG). To solid Boc-protected amine **3** (338 mg, 0.67 mmol) was added concentrated HCl solution in EtOAc (20 mL), prepared by bubbling HCl gas through EtOAc for 1 h, and the resulting mixture was vigorously stirred at room temperature for 3 h. The suspension was diluted with Et_2O (100 mL). The solid was separated, then washed with Et_2O (2 x 50 mL) and dried *in vacuo* to afford amine hydrochloride *trans*-**ATG** (281 mg, 95%) as an orange solid.

$[\alpha]_D^{22}$ +20.2 (*c* 0.27, MeOH); decomp. 171 °C; UV/Vis: λ_{max} (5% DMSO in water) = 331 nm; IR (ATR): 2903 (br), 2612 (br), 1726, 1596, 1507 cm^{-1}; δ_H (600 MHz, CD_3OD) 8.48 (s, 1H), 8.13 (d, *J* = 8.7 Hz, 2H), 8.06 (d, *J* = 8.9 Hz, 2H), 7.96 (dd, *J* = 8.1, 1.5 Hz, 2H), 7.60 – 7.53 (m, 3H), 4.15 (dd, *J* = 8.1, 6.0 Hz, 1H), 3.30 – 3.22 (m, 1H), 3.20 – 3.12 (m, 2H), 2.48 – 2.41 (m, 1H), 2.05 (ddd, *J* = 14.7, 8.1, 4.6 Hz, 1H); δ_C (150 MHz, CD_3OD) 176.5, 171.4, 153.9, 153.6, 146.5, 139.9, 132.8, 130.4, 125.3, 124.0, 122.7, 122.0, 52.4, 42.4, 32.7, 28.5; HRMS (ESI): calculated for $C_{20}H_{20}N_6O_4$: 409.1619 $[M+H]^+$, found: 409.1618 $[M+H]^+$.

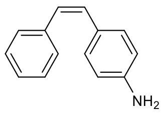

S5

(*Z*)-4-styrylaniline (S5). (*Z*)-1-nitro-4-styrylbenzene (**S4**)[5] (225 mg, 1.00 mmol) was dissolved in EtOH (6.0 mL) and CH_2Cl_2 (6.0 mL), followed by addition of Pd/C (20 mg) and cooling to 0 °C. $N_2H_4 \cdot H_2O$ (85%, 38 μL, 1.00 mmol) was then added dropwise under vigorous stirring and the resulting mixture was warmed to room temperature overnight. After 15 h, the mixture was filtered through a pad of celite with CH_2Cl_2 (30 mL). Concentration *in vacuo* gave (*Z*)-4-styrylaniline **S5** (196 mg, 1.00 mmol, 100%) as a yellow oil. The spectral data matched the one provided in the literature.[6]

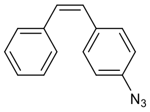

4

(*Z*)-1-azido-4-styrylbenzene (4). Amine **S5** (293 mg, 1.50 mmol) was dissolved in MeCN (4 mL) and cooled to 0 °C before *t*-butyl nitrite (267 μL, 2.25 mmol) was added dropwise, immediately followed by trimethylsilyl azide (239 μL, 1.80 mmol). The reaction mixture was stirred at 0 °C for 1 h, then warmed to room temperature and concentrated *in vacuo* to give a dark-red oil. Column chromatography (hexanes:EtOAc 50:1) gave azide **4** (203 mg, 0.92 mmol, 61%) as a yellow oil.

R_f = 0.35 (hexanes:EtOAc 9:1); ^{1}H NMR (400 MHz, C_6D_6) δ = 7.23 – 7.19 (m, 2H), 7.08 – 7.04 (m, 2H), 7.03 (m, 2H), 7.01 (m, 2H), 6.52 (d, *J* = 8.5 Hz, 2H), 6.44 (d, *J* = 12.2 Hz, 1H), 6.33 (d, *J* = 12.1 Hz, 1H); ^{13}C NMR (101 MHz, C_6D_6) *δ* = 139.2, 137.6, 134.2, 130.7, 130.6, 129.6, 129.2, 128.7, 127.6, 126.9, 119.5, IR (ATR): 3213 (w), 3054 (w), 2924 (m), 2412 (w), 2093 (s), 1599 (m), 1503 (s), 1285 (s), 1244 (s), 1181 (m), 832 (s), 696 (s) cm^{-1}; HRMS (EI): calculated for $C_{14}H_{11}N_3$: 221.0953 M^+, found: 221.0957 M^+.

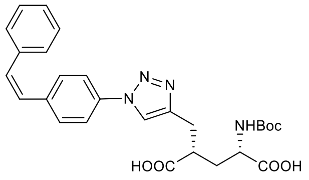

5

(2*S*,4*S*)-2-((*tert*-Butoxycarbonyl)amino)-4-((1-(4-((*Z*)-styryl)phenyl)-1*H*-1,2,3-triazol-4-yl)methyl)pentanedioic acid (5). Alkyne **1** (57 mg, 0.20 mmol) and azide **4** (44 mg, 0.20 mmol) were dissolved in a mixture of MeOH (3 mL), H_2O (1 mL) and DMSO (1 mL) to give a yellow solution. Copper(II) sulfate pentahydrate (10 mg, 0.04 mmol) and sodium ascorbate (10 mg, 0.05 mmol) were added in one portion, and the resulting suspension was stirred at room temperature for 72 h, then quenched with aq. HCl (1 N, 10 mL) and extracted with EtOAc (3 x 40 mL). The organic phase was washed with aq. LiCl (10%, 3 x 70 mL) and brine (100 mL), then dried ($MgSO_4$), filtered and concentrated *in vacuo* to give a colorless foam. Column chromatography (CH_2Cl_2:MeOH:AcOH:H_2O 90:10:0.6:0.6) gave **5** (82 mg, 0.16 mmol, 81%) as a colorless oil, along with 10% reisolated starting materials.

$[\alpha]_D^{22}$ +8.5. (*c* 1.4, MeOH); R_f = 0.34 (CH_2Cl_2:MeOH:AcOH:H_2O 90:10:0.6:0.6); ^{1}H NMR (400 MHz, CD_3OD) δ = 8.26 (s, 1H), 7.77 – 7.62 (m, 2H), 7.42 – 7.32 (m, 2H), 7.29 – 7.15 (m, 5H), 6.73 (d, *J* = 12.2 Hz, 1H), 6.65 (d, *J* = 12.2 Hz, 1H), 4.25 – 4.10 (m, 1H), 3.13 (dt, *J* = 11.0, 5.4 Hz, 1H), 3.09 – 2.89 (m, 2H), 2.25 (ddd, *J* = 13.8, 9.5, 3.8 Hz, 1H), 1.85 (ddd, *J* = 13.5, 10.3, 3.7, Hz 1H), 1.42 (s, 9H), 1.35 – 1.21 (m, 1H); ^{13}C NMR (101 MHz, CD_3OD) δ = 177.8, 176.1, 158.1, 146.9, 139.4, 138.3, 137.0, 132.8, 131.3, 129.9, 129.8, 129.5, 128.5, 122.1, 121.1, 80.5, 53.4, 43.4, 34.5, 29.3, 28.7; IR (ATR): 3213 (w), 3054 (w), 2924 (m), 2515 (w), 1702 (s), 1516 (m), 1446 (s), 1231 (s), 1161 (s), 1023 (m), 783 (s), 699 (s) cm^{-1}; HRMS (ESI): calculated for $C_{27}H_{29}N_4O_6^-$: 505.2093 [M−H]$^-$, found: 505.2084 [M−H]$^-$.

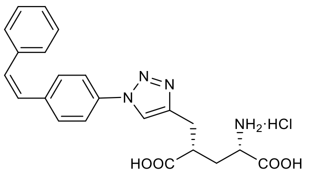

cis-**STG**

(1*S*,3*S*)-1,3-Dicarboxy-4-(1-(4-((*Z*)-styryl)phenyl)-1*H*-1,2,3-triazol-4-yl)butan-1-aminium chloride (*cis*-STG). Boc-Protected *cis*-**STG** (**5**) (21 mg, 41 µmol) in EtOAc (0.5 mL) was treated with a solution of concentrated HCl in EtOAc (2 mL), prepared by bubbling HCl gas through anhydrous EtOAc for 40 min. The turbid solution was stirred for 2 h at room temperature. Upon addition of ice-cold Et_2O (5 mL), a colorless precipitate formed, which

was centrifuged with Et_2O (3 x 10 mL) and dried *in vacuo* to give *cis*-**STG** (14 mg, 31 µmol, 80%) as a colorless solid.

$[\alpha]_D^{22}$ +17.9 (*c* 0.96, MeOH); R_f = 0.45 (MeCN:H_2O 5:1).^{1}H NMR (400 MHz, CD_3OD) δ = 8.41 (s, 1H), 7.70 (d, J = 8.6 Hz, 2H), 7.41 (d, J = 8.5 Hz, 2H), 7.31 – 7.13 (m, 5H), 6.76 (d, J = 12.2 Hz, 1H), 6.68 (d, J = 12.2 Hz, 1H), 4.14 (dd, J = 8.0, 5.9 Hz, 1H), 3.28 – 3.21 (m, 1H), 3.21 – 3.05 (m, 2H), 2.42 (ddd, J = 14.6, 8.5, 6.0 Hz, 1H), 2.02 (ddd, J = 14.6, 8.2, 4.3 Hz, 1H).;^{13}C NMR (101 MHz, CD_3OD) δ = 176.4, 171.3, 146.0, 139.8, 138.2, 136.7, 133.0, 131.4, 129.9, 129.7, 129.5, 128.5, 123.0, 121.3, 121.2, 52.4, 42.4, 32.6; HRMS (ESI): calculated for $C_{22}H_{22}N_4O_4Na$: 429.1533 $[M+Na]^+$, found: 429.1539 $[M+Na]^+$; IR (ATR): 3376 (br), 2922 (s), 2515 (br), 1918 (br w), 1720 (s), 1599 (m), 1513 (s), 1446 (m), 1222 (br s), 832 (s), 698 (s) cm^{-1}.

Supplementary references

1. Volgraf, M.; Gorostiza, P.; Szobota, S.; Helix, M. R.; Isacoff, E. Y.; Trauner, D., *J. Am. Chem. Soc.* **2007,** *129*, 260-261.

2. Sekhri, L.; Bebba, A. A.; Hassini, Z., *Asian J. Chem.* **2005,** *17*, 2455-2462.

3. Troger, J.; Hille, W.; Vasterling, P., *J. Prakt. Chem.* **1905,** *72*, 511-535.

4. Barral, K.; Moorhouse, A. D.; Moses, J. E., *Org. Lett.* **2007,** *9*, 1809-1811.

5. R. Antonioletti, F. Bonadies, A. Ciammaichella, A. Viglianti, *Tetrahedron* **2008**, *64*, 4644-4648.

6. C. Belger, B. Plietker, *Chem. Commun.* **2012**, *48*, 5419-5421.
